# Effects of yeast culture and oxalic acid supplementation on *in vitro* nutrient disappearance, rumen fermentation, and bacterial community composition

**DOI:** 10.3389/fvets.2023.1330841

**Published:** 2024-01-19

**Authors:** Natnael D. Aschalew, Longyu Zhang, Ziyuan Wang, Yuanhong Xia, Guopei Yin, Jianan Dong, Yuguo Zhen, Xuefeng Zhang, Tao Wang, Zhe Sun, Guixin Qin

**Affiliations:** ^1^Key Laboratory of Animal Nutrition and Feed Science of Jilin Province, Key Laboratory of Animal Production Product Quality and Security Ministry of Education, JLAU-Borui Dairy Science and Technology R&D Center, College of Animal Science and Technology, Jilin Agricultural University, Changchun, China; ^2^College of Agriculture and Environmental Science, Dilla University, Dilla, Ethiopia; ^3^Postdoctoral Scientific Research Workstation, Feed Engineering Technology Research Center of Jilin Province, Changchun Borui Science and Technology Co., Ltd., Changchun, China; ^4^College of Life Sciences, Engineering Research Center of Bioreactor and Pharmaceutical Development, Ministry of Education, Jilin Agricultural University, Changchun, China

**Keywords:** rumen fermentation, bacterial community composition, *in vitro* disappearance, oxalic acid, volatile fatty acids, yeast culture

## Abstract

Hemicellulose is an important polysaccharide in ruminant nutrition, but it has not been studied as thoroughly as cellulose. Further research is needed to explore supplements that can improve its digestibility and ruminal buffering effects. Our previous research demonstrated the efficacy of oxalic acid (OA) as an essential nutrient in yeast culture (YC) for improving rumen fermentation performance. Consequently, we conducted *in vitro* rumen digestion experiments to examine the effects of YC and OA on rumen fermentation and bacterial composition. Two diets containing different levels of hemicellulose were formulated: diet 1 with 10.3% and diet 2 with 17% hemicellulose. Three levels of YC (0.00, 0.625, and 1.25 g/kg) and three doses of OA (0.0, 0.4, and 0.8 g/kg, DM) were added into each diet with a 3 × 3 factorial design. A comprehensive assessment was conducted on a total of 18 experimental treatments at fermentation periods of 0, 6, 12, 24, and 48 h. In the first experiment (diet 1), the supplementation of YC, OA, and their interaction significantly increased *in vitro* DM disappearance (IVDMD) and NDF disappearance (IVNDFD; *p* < 0.001). In the second experiment (diet 2), the supplementation of OA and the interaction between YC and OA (*p* < 0.001) increased IVDMD and IVCPD, but had no significant effects on IVNDFD. The interactions of YC and OA significantly increased ammonia nitrogen (*p* < 0.001). The production of acetic acid, propionic acid, and total volatile fatty acids (TVFA), and pH levels were significantly higher in treatments supplemented with YC and OA (*p* < 0.001). YC and OA in both diets significantly altered the rumen bacterial community leading to increased Shannon and Simpson diversity indices (*p* < 0.001). In both diets, OA supplementation significantly increased the relative abundance of the phylum *Bacteroidetes* and *Prevotella* genus. The result also showed a positive correlation between the *Prevotella* and *Selenomonas* genera with IVDMD, IVNDFD, propionic acid, and TVFA production, suggesting that these dominant bacteria enhanced nutrient disappearance in the rumen. In conclusion, adding YC and OA resulted in modifications to the bacterial community’s composition and diversity, and improved nutrient disappearance. These changes indicate improved rumen fermentation efficiency, which is promising for future *in vivo* studies.

## 1 Introduction

The increasing demand for intensive sheep production has led to the strategic use of expensive feed ingredients to boost productivity. As a result, producers are providing grains, grain processing byproducts, and concentrates to their sheep in order to achieve higher returns (1). This primarily impacts the animals’ health by reducing the pH levels in their rumen, resulting in subacute ruminal acidosis and other metabolic disorders. In response, feed manufacturing companies have recently begun incorporating corn bran into the total mixed ration (TMR) due to its high hemicellulose content (2), as it is the richest source of hemicellulose.

Hemicellulose is a polysaccharide comprising all cell wall constituents except cellulose and pectin (3), that plays a significant role in ruminant nutrition as an important component of fiber, but it has not been as extensively researched as cellulose. Anaerobic microorganisms, including hemicellulolytic and nonhemicellulolytic microbes, as well as extracellular enzymes can break down and ferment hemicellulose in the rumen to produce volatile fatty acids (VFA) and stabilize the rumen ecosystem of the host (4). Currently, there is a lack of research on the effectiveness of supplements in enhancing hemicellulose digestibility and ruminal buffering effects. Consequently, in order to enhance hemicellulose digestion, it is necessary to design and implement other alternatives.

*Saccharomyces cerevisiae* is a unicellular fungus commonly added to animal feed as yeast culture (YC) or live yeast (LY) supplement that facilitates carbohydrate fermentation (5). Adding YC to the ruminant’s diet can stimulate fiber digestion (6) and change fermentation by improving the rumen microflora composition (7, 8), but it is not a nutrient source (9). Plata and Bárcena-Gama (10) conducted a study showing that adding YC to the diet could facilitate the breakdown of hemicellulose in the rumen. On the other hand, studies have shown that adding YC to the diet can reduce ammonia nitrogen concentration (11, 12) and rumen microbiome (13).

Oxalic acid (OA) is a low molecular weight organic acid that is synthesized by several organisms, including fungi, bacteria, plants, and animals (14, 15) and is most abundantly found in the environment (14). Roughage feeds supplemented with organic acids improved rumen fermentation efficiency and fiber degradation (16, 17). Other researchers found that OA decreased DM digestibility, ammonia nitrogen concentration, and butyrate production and had a negative impact on rumen microbial activities (18). However, it is still being produced sufficiently in the environment (14, 15, 19). Therefore, finding options to effectively use this potentially available organic acid especially as a feed supplement is important for livestock producers, feed processing companies and reagents manufacturing industries. Furthermore, we previously reported that oxalic acid (OA) plays a crucial role in enhancing rumen fermentation and nutrient digestion in YC. Consequently, the supplementation levels of YC and OA were determined based on the recommendations derived from these findings (20). Hence, this research hypothesized that combining YC and OA may enhance rumen fermentation efficiency, nutrient digestion, and alter bacterial community composition. Therefore, two 3 × 3 factorial *in vitro* experiments were designed to examine the effects of YC and OA on nutrient disappearance, rumen fermentation parameters, and bacterial community composition.

## 2 Materials and methods

### 2.1 Animals

Four short-tailed, male, Han sheep (average live weight, 36 ± 3.52 kg, *mean ± SD*) were used as donors of rumen fluid. The sheep were fitted with a round-shaped and flexible rumen cannula. They were fed pelleted concentrate and oat hay in a 1:1 concentrate to forage ratio twice daily at 07:30 AM and 05:30 PM and had free access to fresh, clean water.

### 2.2 Experimental treatment formulation and design

Based on the fiber requirements of sheep (21) specifically neutral detergent fiber (NDF) and acid detergent fiber (ADF) levels, two hemicellulosic diets were formulated. These diets were named diet 1 and diet 2, their hemicellulose levels were 10.3% and 17%, respectively. The two diets were supplemented with 3 levels of YC (0.000, 0.625, and 1.250 g/kg) and 3 doses of OA (0.0, 0.4, and 0.8 g/kg of feed, DM basis) in a 3 × 3 factorial design. A total of 18 treatments (9 in each diet) were designed and evaluated. The YC and OA levels are expressed as grams per kilogram of feed on a dry matter basis. Table 1 presents detailed information about the experimental treatments in both diets. The ingredients and nutritional chemical composition of the two diets are shown in Table 2. The YC was manufactured under the controlled microenvironment of the JLAU-Borui Dairy Science and Technology R&D Center, and the oxalic acid was purchased from the RHAWN reagents supplying company (Shanghai, China).

**Table 1:** The experimental treatments with levels of supplements used in the study.

Diets	Treatments	Yeast culture levels (g/kg)	Oxalic acid levels (g/kg)
Diet 1 (10.3% hemicellulose)	C	-	-
	y	0.625	-
	Y	1.25	-
	o	-	0.40
	O	-	0.80
	yo	0.625	0.40
	yO	0.625	0.80
	Yo	1.25	0.40
	YO	1.25	0.80
Diet 2 (17% hemicellulose)	ctrl	-	-
	L	0.625	-
	H	1.25	-
	l	-	0.40
	h	-	0.80
	Ll	0.625	0.40
	Lh	0.625	0.80
	Hl	1.25	0.40
	Hh	1.25	0.80

Where: C = Diet 1 (10.3% hemicellulose); y = diet 1 with low yeast culture; Y = Diet 1 with high yeast culture; o = diet 1 with low oxalic acid; O = diet 1 with high oxalic acid; yo = diet 1 with low yeast culture and low oxalic acid; yO = diet 1 with low yeast culture and high oxalic acid; Yo = diet 1 with high yeast culture and low oxalic acid; YO = diet 1 with high yeast culture and high oxalic acid; ctrl = Diet 2 (17% hemicellulose); L = diet 2 with low yeast culture; H = diet 2 with high yeast culture; l = diet 2 with low oxalic acid; h = diet 2 with high oxalic acid; Ll = diet 2 with low yeast culture and low oxalic acid; Lh = diet 2 with low yeast culture and high oxalic acid; Hl = diet 2 with high yeast culture and low oxalic acid; and Hh = diet 2 with high yeast culture and high oxalic acid. The hemicellulose, yeast culture, and oxalic acid levels are expressed on a dry matter basis.

**Table 2:** Ingredients and nutritional composition of the experimental diets (%, DM).

Item	Diet 1 (10.3% hemicellulose)	Diet 2 (17% hemicellulose)
**Ingredients**		
Corn	38.12	35.71
Soybean meal	16.18	13.27
Corn germ meal (spray)	6.18	6.12
Corn bran	10.30	31.63
Peanut shell	15.46	0.00
Corn oil	0.36	0.00
Bentonite	4.12	4.08
Sugar	4.12	4.08
Premix^a^	5.15	5.10
Total	100	100
**Nutritional compositions (% DM)**		
Dry matter	90.5	90.3
Digestible energy^b^ MJ/kg	11.6	12.2
Crude protein	13.8	13.9
Crude fat	2.7	2.8
Crude fiber	13.1	6.7
Ash	14.6	12.4
Starch	30.1	30.3
Neutral detergent fiber	26.0	25.8
Acid detergent fiber	15.7	8.8
Hemicellulose^c^	10.3	17
Calcium	0.6	0.6
Phosphorus	0.4	0.4

Where: ^a^Premix composition (per kilogram): FeSO_4_, 8,453 mg; CuSO_4._5H_2_O, 1,480 mg; MnSO_4_, 13,241 mg; ZnSO_4_.5H_2_O, 8,294 mg; CoCl_2_, 16 mg; KI, 30 mg; Na_2_SeO_3_ (1% Se), 377 mg; vitamin A, 755 IU; vitamin D, 113 IU; vitamin E, 887 IU. ^b^Calculated based on Ruminants (21). ^c^Calculated by the difference between NDF and ADF.

### 2.3 Nutritional chemical composition analyses

The dried feed samples were ground to pass through a 1 mm screen using a Wiley mill (Thomas Scientific, Swedesboro, NJ) for all nutritional composition analyses. The chemical composition was determined using AOAC (22) methods: method 934.01 for DM, method 942.05 for ash, and method 984.13 for N determination. The acid detergent fiber (ADF) and neutral detergent fiber (NDF) contents were evaluated using the ANKOM^200^ Fiber Analyzer (ANKOM Technology, Macedon, NY, United States). Reagents such as sodium sulfite and α-amylase (ANKOM Technology) were used in the NDF analysis, while sulfuric acid was used for ADF extraction. The hemicellulose level was computed using the difference between the NDF and ADF levels.

### 2.4 *In vitro* nutrient disappearance

To measure the nutrient disappearance, an ANKOM DAISY^II^ incubator (ANKOM Technology, Macedon, NY, United States) was used. Before the morning feeding, the rumen fluid was collected from four rumen-cannulated sheep and filtered through four layers of cheesecloth. Subsequently, a total volume of 1,200 mL of buffer solution (23) and 400 mL of rumen fluid were added to each digestion jar. One gram of experimental treatment was measured and added to the F-57 ANKOM filter bag. An empty F-57 filter bag was included in each phase for the correction factor. The filter bags were heat-sealed and placed directly into the digestion jars. The jars were purged with CO_2_ for 30 s to create an anaerobic environment, then tightly covered and incubated for 0, 6, 12, 24, and 48 h in an ANKOM DAISY^II^ incubator. Each treatment was replicated 15 times (5 replications for each parameter) at each fermentation time to measure *in vitro* dry matter (IVDMD), neutral detergent fiber (IVNDFD), and crude protein (IVCPD) disappearance.

A total of 1,350 ANKOM F-57 filter bags were used for measuring nutrient disappearance. After fermentation, each bag was rinsed with cold tap water until the water ran clear and was agitated to remove any remaining water. Then, each bag was air-dried and oven dried at 55°C for 72 h with bags being turned upside down every 12 h to ensure uniform drying. The weight of each dried bag was then measured. At each fermentation time, some bags were used for further NDF and CP analyses using the methods mentioned in section 2.3. Finally, the following mathematical formulas were used to calculate *in vitro* nutrient disappearance (24, 25).


$$\text{IVDMD}\;\left(\%\;\text{of}\;DM\right)=\;\left(\frac{\begin{array}{l} \text{Initial}\;\text{Sample}\;DM\;\text{weigh}\;\left(g\right)\\ -\;\text{Sample}\;\text{weight}\;\text{residue}\;\left(g\right) \end{array}}{\text{Initial}\;\text{Sample}\;DM\;\text{weight}\;\left(g\right)}\right)\times 100$$

$$\text{IVNDFD}\;\left(\%\;\text{of}\;NDF\right)=\left(\frac{\begin{array}{l} NDF\;\text{before}\;\text{fermentation}\;\left(g\right)\\ -\;NDF\;\text{residue}\;\left(g\right) \end{array}}{NDF\;\text{before}\;\text{fermentation}}\right)\times 100$$

$$\text{IVCPD}\;\left(\%\;\text{of}\;CP\right)=\left(\frac{\begin{array}{l} CP\;\text{before}\;\text{fermentation}\left(g\right)\\ -\;CP\;\text{residue}\;\left(g\right) \end{array}}{CP\;\text{before}\;\text{fermentation}\;\left(g\right)}\right)\times 100$$

### 2.5 Rumen fermentation parameters

All samples used to measure *in vitro* ruminal efficiency parameters such as volatile fatty acids (VFA), ammonia nitrogen (NH_3_-N), and ruminal microbiome composition were processed using the ANKOM*^RF^* gas production system. Two grams of the experimental treatment were added to 250 mL ANKOM bottles. The mixed buffer solution and rumen fluid were purged with CO_2_ at each step, added to the sample-containing bottles, and placed inside an incubator shaker with an internal temperature of 39°C and a speed of 80 rpm (Tianjin Honor Instrument Shaker Co. Ltd., Tianjin, China). After each incubation time (0, 6, 12, 24, 48 h) was completed, the pH values were immediately measured. Then, a 2 mL aliquot of fluid was collected and preserved at −20°C until VFA and NH_3_-N tests were performed. The samples for microbial composition analysis were stored at −80°C. Specific procedures for each rumen fermentation parameter are detailed below.

#### 2.5.1 Ruminal fluid pH

The ruminal fluid pH was measured with a Sanxin MP523-04 digital pH meter (Shanghai Sanxin Instrumentation, Inc., Shanghai, China) according to the manufacturer’s guidelines.

#### 2.5.2 Volatile fatty acids analysis

The analysis of volatile fatty acids (VFA) was carried out using an Agilent 7,890 A gas chromatograph (Agilent Technologies, Santa Clara, California, United States) and a 50 m (internal diameter 0.32 mm) CP-Wax Chrompack silica-fused capillary column (Varian, Palo Alto, California, United States). The initial and final oven temperatures were set at 65 and 195°C, respectively, with helium used as the carrier gas. The detector and injector temperatures were carefully calibrated to 250°C, and the injector volume was precisely configured to 1 μL. The samples were centrifuged at 10,000 × *g* at 4°C for 10 min. The centrifuged sample was then transferred to another 2 mL EP tube, and 0.2 mL of metaphosphoric acid was added. The mixture was refrigerated for approximately 3 h, then removed and centrifuged again at 10,000 × *g* at 4°C for 10 min to obtain the supernatant. A 1 mL of the supernatant sample was placed into the GC beaker and processed by gas chromatography.

#### 2.5.3 Ammonia nitrogen concentration

The NH_3_-N concentration was measured using the Chaney and Marbach (26) method, with a Cary Series UV–Vis Spectrophotometer connected to a computer running Cary Series analysis software (Agilent Technologies, California, United States). In detail, the samples were centrifuged at 4,000 × *g* for 10 min at 4°C, and 0.2 mL of supernatant was combined with 1.8 mL of distilled water and 8 mL of 0.2 M hydrochloric acid in 15-mL centrifuge tubes. From each 15-mL tube, 0.4 mL was transferred to a 10-mL tube, and 2 mL of each solution A and B were added. Lastly, analyzed by a Cary Series UV–Vis Spectrophotometer. The measured extinction values were substituted into the regression formula, and the results were multiplied by a dilution factor to determine the final NH_3_-N concentration in the sample.

#### 2.5.4 Cumulative gas production

The ANKOM^RF^ gas production system is designed to measure the gas pressure in psi. The gas production for each bottle was measured and recorded over time. The data was then converted into moles of gas using the ‘ideal’ gas law and further converted into milliliters (mL) of gas using Avogadro’s law.

The ideal gas law:


$$n=p\left(\frac{V}{RT}\right)$$

where: n—is the quantity of gas generated, measured in moles (mol).

p—pressure in kilo pascal (kPa).
V—the glass bottle’s head-space volume, expressed in liters (L).
T—the internal temperature in Kelvin (K).
R—gas constant (8.314472 L·kPa·K^−1^·mol^−1^).

Avogadro’s law states that, under standard conditions, 1 mole will fill 22.4 L at 273.15°K and 101.325 kPa (1 psi = 6.894757293 kilopascal). Therefore, the following formula can be used to convert a gas mole measurement to milliliters (ANKOM manual):


$$\text{Gas}\;\text{produced}\;\left(\text{mL}\right)=n\times 22.4\times 1000$$

### 2.6 Rumen microbiota composition analysis

#### 2.6.1 DNA sequencing

Sequencing, library preparations, and taxonomic analysis were performed by Shanghai Personal Biotechnology Co. Ltd. (Shanghai, China). A total of 216 ruminal fluid samples produced 30,196,306 original sequences, with 19,625,098 (90,857 sequences per sample) identified as high-quality bacterial sequences. The 16S ribosomal RNA (rRNA) gene was amplified using the polymerase chain reaction (PCR) method. Barcoded tags and the widely used bacterial primers 341F (5′-CCTAYGGGRBGCASCAG-3′) and 806R (5′-GGACTACNNGGGTATCTAAT-3′) were used in the amplification process (27). The V4 region of the gene was amplified from genomic DNA and the resulting amplicons were prepared for sequencing using Illumina’s MiSeq technology (28).

#### 2.6.2 Taxonomic composition analysis

Based on the distribution of OTU in different samples, the levels of alpha diversity in each sample were assessed. An association network was constructed for each sample, and the topological index was calculated to identify the key species. The composition and distribution of each sample at phylum, class, order, family, genus, and species levels were analyzed visually and statistically using QIIME2 in the R programming language. This analysis involved removing singleton data points and statistically summarizing all the information in a table. To fully evaluate the richness of the microbial community alpha diversity, the Chao1 (29) index, as well as the Shannon (30) and Simpson (31) indices, were used and analyzed. Additionally, Principal Coordinates Analysis (PCoA) of beta diversity was conducted to compare the distance between the two samples.

### 2.7 Statistical data analysis

The MIXED procedure of SAS (version 9.4; SAS Institute Inc., Cary, NC) was used to analyze all variables. For statistical analyses, treatments in the two hemicellulosic diets were analyzed and computed separately. A two-way ANOVA was used, and the sources of variation between treatments were taken as fixed effect variables and incubation time as a random effect. The significance level was declared at *p* ≤ 0.05 for Duncan’s New Multiple Range Test (MRT), which was used to examine the significant variables for multiple comparisons of the mean values. Microbiome community composition data were analyzed using several tools inside R programming language software (32), and the model for both experiments was as follows:


$$\text{Yijk}=\mu +Yi+Oj+\left(YO\right)ij+\text{eijk}$$

Where: Y_ijk_ = the dependent variables; μ = overall mean; Y_i_ = yeast culture effect (_i_ = 1–3); O_j_ = oxalic acid effect (_j_ = 1–3); (YO)_ij_ = effect due to YC and OA interaction; and e_ijk_ = random residual error.

## 3 Results

### 3.1 *In vitro* nutrient disappearance

**Figure 1:**
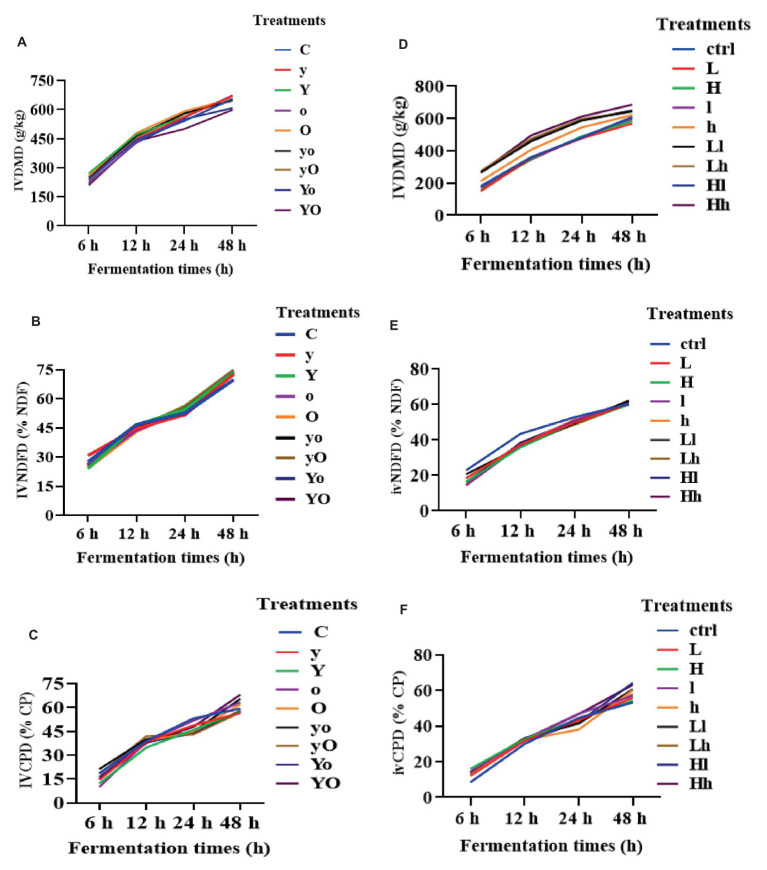
The *in vitro* nutrient disappearance of diet 1 treatments DM **(A)**, NDF **(B)**, CP **(C)**, and diet 2 **(D–F)** respectively. Where: C = Diet 1 (10.3% hemicellulose); y = diet 1 with low yeast culture; Y = diet 1 with high yeast culture; o = diet 1 with low oxalic acid; O = diet 1 with high oxalic acid; yo = diet 1 with low yeast culture and low oxalic acid; yO = diet 1 with low yeast culture and high oxalic acid; Yo = diet 1 with high yeast culture and low oxalic acid; YO = diet 1 with high yeast culture and high oxalic acid; ctrl = Diet 2 (17% hemicellulose); L = diet 2 with low yeast culture; H = diet 2 with high yeast culture; l = diet 2 with low oxalic acid; h = diet 2 with high oxalic acid; Ll = diet 2 with low yeast culture and low oxalic acid; Lh = diet 2 with low yeast culture and high oxalic acid; Hl = diet 2 with high yeast culture and low oxalic acid; Hh = diet 2 with high yeast culture and high oxalic acid. IVDMD = *in vitro* dry matter disappearance; IVNDFD = *in vitro* neutral detergent fiber disappearance; IVCPD = *in vitro* crude protein disappearance.

Figure 1 illustrates the changes that occurred in the amounts of *in vitro* dry matter (IVDMD), neutral detergent fiber (IVNDFD), and crude protein (IVCPD). In diet 1, supplementation with YC, OA, and their interaction increased IVDMD at 24 and 48 h (*p* < 0.001; Figure 1A). At 48 h, YC (*p* = 0.05), OA (*p* < 0.01), and their combinations (*p* < 0.01) also increased IVNDFD (Figure 1B). Additionally, at 48 h, OA (*p* < 0.05) and the YC and OA interactions (*p* < 0.01) significantly increased IVCPD (Figure 1C). In diet 2, YC, OA, and their interaction significantly increased IVDMD (*p* < 0.001; Figure 1D). YC and OA supplementation reduced IVNDFD at 12 h (*p* < 0.001; Figure 1E). Furthermore, all supplemented treatments showed higher IVCPD than the control (*p* < 0.001; Figure 1F). These results indicated that YC, OA, and their interactions may enhance *in vitro* nutrient disappearance.

### 3.2 Rumen fermentation parameters

**Figure 2:**
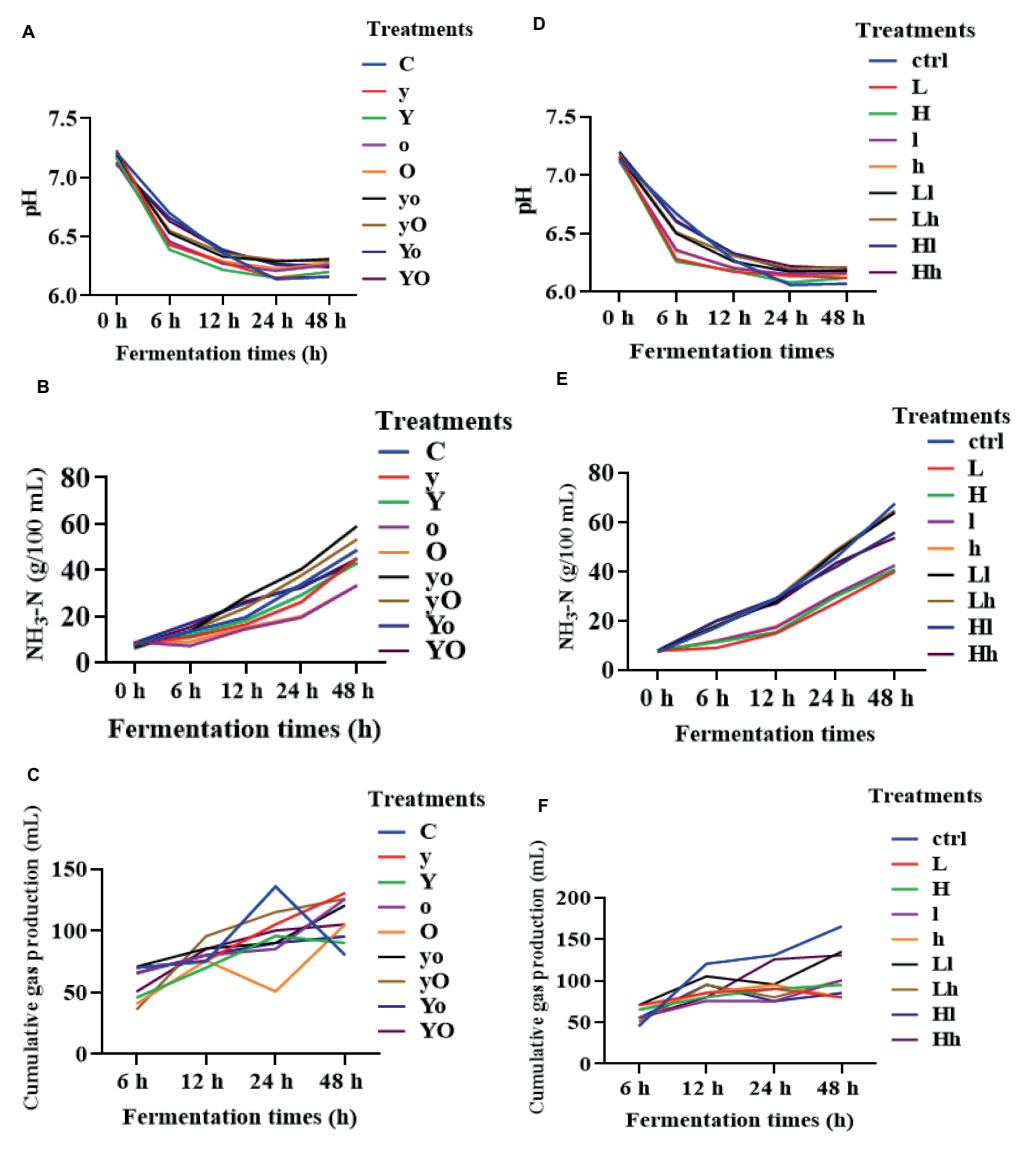
Rumen fermentation parameters of diet 1 pH **(A)**, NH_3_-N **(B)**, cumulative gas production **(C)**, and diet 2 **(D–F)** respectively. Where: C = Diet 1 (10.3% hemicellulose); y = diet 1 with low yeast culture; Y = diet 1 with high yeast culture; o = diet 1 with low oxalic acid; O = diet 1 with high oxalic acid; yo = diet 1 with low yeast culture and low oxalic acid; yO = diet 1 with low yeast culture and high oxalic acid; Yo = diet 1 with high yeast culture and low oxalic acid; YO = diet 1 with high yeast culture and high oxalic acid; ctrl = Diet 2 (17% hemicellulose); L = diet 2 with low yeast culture; H = diet 2 with high yeast culture; l = diet 2 with low oxalic acid; h = diet 2 with high oxalic acid; Ll = diet 2 with low yeast culture and low oxalic acid; Lh = diet 2 with low yeast culture and high oxalic acid; Hl = diet 2 with high yeast culture and low oxalic acid; Hh = diet 2 with high yeast culture and high oxalic acid; NH_3_-N = ammonia nitrogen.

In diet 1, YC, OA, and their combination significantly increased the ruminal fluid pH (Figure 2A). The pH value of the control was lower than that of the supplemented treatments at 24 and 48 h (*p* < 0.05). The YC and OA interactions also significantly increased NH_3_-N (*p* < 0.001), while individual supplementation with either YC or OA reduced the NH_3_-N concentration (Figure 2B). Additionally, the YC and OA interactions reduced the cumulative gas production at 24 h (*p* = 0.05; Figure 2C). In diet 2, individual supplementation of OA significantly increased the ruminal fluid pH (*p* < 0.001; Figure 2D). The YC and OA interactions also increased the NH_3_-N concentration at 24 h (*p* < 0.001; Figure 2E).

In diet 1, the supplemented groups showed significantly higher levels of acetic acid (*p* < 0.05, 12 h), TVFA (*p* < 0.001, 24 and 48 h), and propionic acid (*p* < 0.001; Table 3). In diet 2, treatments supplemented with OA produced higher levels of acetic acid compared to YC alone and their interaction (*p* < 0.001). Table 4 indicates that treatments supplemented with lower levels of OA, and both lower and higher levels of YC increased TVFA production (*p* < 0.05). additionally, supplementation with YC and OA in this diet significantly reduced the cumulative gas production during fermentation (Figure 2F).

**Table 3:** Effects of YC and OA supplementation in diet 1 on major VFA production (mmol/L).

Item	Time	Diet 1	Yeast culture (YC)		Oxalic acid (OA)		Interactions				SEM	*p* value		
		C	y	Y	o	O	yo	yO	Yo	YO		YC	OA	YC × OA
Acetic acid	0 h	15.80	15.22	14.45	14.77	15.40	15.89	17.20	16.43	16.75	0.31	0.593	0.268	0.494
	6 h	33.14^ab^	32.21^ab^	31.83^a^	41.20^d^	38.66^cd^	33.32^ab^	31.57^a^	33.39^ab^	35.94^bc^	0.66	<0.001	<0.001	<0.001
	12 h	44.01^ab^	39.97^ab^	38.24^a^	43.00^ab^	46.46^b^	43.31^ab^	40.93^ab^	42.17^ab^	43.24^ab^	0.58	0.012	0.049	0.157
	24 h	58.53^e^	44.97^ab^	44.26^a^	51.30^cd^	53.43^de^	51.21^cd^	47.83^abcd^	50.19^bcd^	47.11^abc^	0.89	<0.001	0.207	<0.001
	48 h	70.95^b^	49.95^a^	56.22^a^	56.37^a^	57.26^a^	57.70^a^	53.02^a^	55.63^a^	54.54^a^	1.16	<0.001	0.018	<0.001
Propionic acid	0 h	7.62	7.47	7.13	7.79	8.05	7.85	8.58	8.59	8.77	0.14	0.513	0.007	0.338
	6 h	14.13^a^	29.89^d^	29.35^d^	34.73^e^	32.72^de^	23.45^c^	22.09^c^	18.02^b^	20.62^b^	1.31	<0.001	<0.001	<0.001
	12 h	18.14^a^	36.98^d^	35.15^d^	37.67^d^	39.93^d^	29.48^c^	27.86^bc^	23.23^ab^	24.38^bc^	1.43	<0.001	0.726	<0.001
	24 h	22.97^a^	41.78^e^	40.79^e^	43.74^ef^	46.19^f^	33.88^d^	30.45^c^	27.50^bc^	26.78^b^	1.57	<0.001	<0.001	<0.001
	48 h	27.47^a^	44.67^d^	50.03^e^	46.59^de^	49.07^de^	36.61^c^	33.02^bc^	30.71^ab^	30.63^ab^	1.66	<0.001	0.003	<0.001
Butyric acid	0 h	3.80	3.75	3.57	3.51	3.75	3.85	4.35	4.04	4.29	0.09	0.331	0.154	0.543
	6 h	9.25^cd^	11.61^e^	11.69^e^	6.94^ab^	6.52^a^	10.04^d^	9.53^d^	7.40^ab^	7.93^bc^	0.36	<0.001	<0.001	0.001
	12 h	13.31^c^	15.12^c^	14.26^c^	9.28^a^	10.05^a^	14.0^5c^	13.28^bc^	10.34^a^	10.69^ab^	0.43	<0.001	<0.001	0.074
	24 h	19.59^d^	17.89 cd	16.99^cd^	12.92^a^	13.24^ab^	17.58^cd^	15.86^bc^	13.91^ab^	12.04^a^	0.51	<0.001	<0.001	<0.001
	48 h	22.81^f^	18.28^cde^	20.84^ef^	14.54^a^	15.59^abc^	19.60^de^	17.53^abc^	15.23^ab^	15.57^abc^	0.55	0.052	<0.001	<0.001
TVFA	0 h	28.75	27.93	26.58	27.35	28.58	29.06	31.75	30.50	31.29	0.56	0.541	0.138	0.467
	6 h	59.68^a^	79.21^cd^	78.53^cd^	87.31^d^	82.08^d^	71.49^bc^	67.47^ab^	62.55^ab^	68.45^ab^	1.80	0.002	0.649	<0.001
	12 h	79.83^a^	99.49^c^	94.79^abc^	95.50^bc^	102.34^c^	93.39^abc^	88.46^abc^	80.56^ab^	83.49^ab^	1.73	0.016	<0.001	0.049
	24 h	108.58^bcd^	114.46^cde^	111.56^cde^	116.04^de^	121.11^e^	111.95^cde^	102.82^abc^	98.77^ab^	92.25^a^	1.81	<0.001	0.018	<0.001
	48 h	131.89^c^	125.13^abc^	143.50^c^	130.49^bc^	133.28^c^	126.03^abc^	114.32^ab^	110.73^a^	110.07^a^	2.19	0.002	<0.001	<0.001
A:P	0 h	2.02	1.95	2.01	1.98	1.91	1.97	1.98	2.03	1.94	0.02	0.88	0.62	0.87
	6 h	2.35^f^	1.08^a^	1.09^a^	1.19^b^	1.18^b^	1.42^c^	1.43^c^	1.85^e^	1.74^d^	0.08	<0.001	<0.001	<0.001
	12 h	2.43^e^	1.08^a^	1.09^ab^	1.14^ab^	1.16^b^	1.47^c^	1.47^c^	1.82^d^	1.78^d^	0.08	<0.001	<0.001	<0.001
	24 h	2.55^d^	1.08^a^	1.09^a^	1.17^a^	1.16^a^	1.51^b^	1.57^b^	1.83^c^	1.76^c^	0.09	<0.001	<0.001	<0.001
	48 h	2.59^d^	1.12^a^	1.12^a^	1.21^a^	1.17^a^	1.58^b^	1.61^b^	1.81^c^	1.78^c^	0.09	<0.001	<0.001	<0.001

^a,b,c,d,e,f^Different superscripts in the same row imply that their mean values are significantly different (*p* ≤ 0.05). Where: C = Diet 1 (10.3% hemicellulose); y = diet 1 with low yeast culture; Y = diet 1 with high yeast culture; o = diet 1 with low oxalic acid; O = diet 1 with high oxalic acid; yo = diet 1 with low yeast culture and low oxalic acid; yO = diet 1 with low yeast culture and high oxalic acid; Yo = diet 1 with high yeast culture and low oxalic acid; YO = diet 1 with high yeast culture and high oxalic acid; TVFA = total volatile fatty acid; A:P = acetic to propionic acid ratio.

**Table 4:** Effects of YC and OA supplementation in diet 2 on major VFA production (mmol/L).

Item	Time	Diet 2	Yeast culture (YC)		Oxalic acid (OA)		Interactions				SEM	*p*-value		
		ctrl	L	H	l	h	Ll	Lh	Hl	Hh		YC	OA	YC × OA
Acetic acid	0 h	16.22	15.70	16.80	16.06	16.81	16.35	15.77	16.37	19.12	0.33	0.185	0.383	0.544
	6 h	35.94^bcd^	30.36^a^	38.23^d^	37.52^cd^	45.88^e^	31.84^a^	31.67^a^	33.05^ab^	34.13^abc^	0.91	<0.001	<0.001	<0.001
	12 h	54.68^d^	37.63^a^	41.60^abc^	45.87^c^	56.74^d^	38.73^ab^	41.00^abc^	44.11^bc^	41.58^abc^	1.29	<0.001	0.004	<0.001
	24 h	58.10^bc^	49.32^a^	49.54^ab^	53.64^ab^	62.92^c^	48.64^a^	50.32^ab^	50.02^ab^	49.63^ab^	1.03	<0.001	0.072	0.119
	48 h	61.79^bc^	57.76^abc^	54.52^a^	62.71^c^	72.10^d^	56.68^abc^	58.18^abc^	55.17^ab^	54.69^a^	1.11	<0.001	0.008	0.006
Propionic acid	0 h	8.71	8.18	8.89	8.55	8.98	8.65	8.32	8.26	9.59	0.155	0.393	0.446	0.538
	6 h	15.49^a^	28.92^d^	35.50^f^	32.56^e^	40.39^g^	22.72^c^	22.54^c^	19.37^b^	9.48^b^	1.57	<0.001	<0.001	<0.001
	12 h	22.51^a^	35.32^d^	38.56^de^	39.86^e^	51.88^f^	26.98^bc^	28.09^c^	25.82^abc^	24.31^ab^	1.78	<0.001	<0.001	<0.001
	24 h	24.15^a^	44.47^c^	44.66^c^	45.68^c^	56.27^d^	32.67^b^	33.38^b^	29.94^b^	29.18^b^	1.94	<0.001	0.002	<0.001
	48 h	26.54^a^	51.24^de^	48.12^d^	53.56^e^	60.69^f^	37.00^c^	38.45^c^	31.97^b^	31.89^b^	2.17	<0.001	0.001	<0.001
Butyric acid	0 h	4.07	3.94	4.45	4.14	4.35	4.26	4.06	4.23	5.20	0.13	0.207	0.429	0.652
	6 h	9.61^bc^	11.68^d^	13.84^e^	6.92^a^	8.75^b^	9.82^c^	9.55^bc^	7.15^a^	7.57^a^	0.42	<0.001	<0.001	<0.001
	12 h	15.60^d^	14.35^cd^	15.36^d^	10.18^a^	13.44^bc^	12.4^b^	13.09^bc^	10.66^a^	9.93^a^	0.41	<0.001	<0.001	<0.001
	24 h	18.90^d^	18.02^d^	18.19^d^	12.81^a^	16.48^bcd^	16.32^bcd^	17.14^cd^	13.68^abc^	13.53^ab^	0.46	0.008	<0.001	0.013
	48 h	20.31^c^	19.45^c^	18.71^bc^	16.39^c^	20.94^c^	8.61^bc^	19.38^c^	15.61^a^	15.20^a^	0.37	<0.001	<0.001	0.007
TVFA	0 h	30.42	29.28	31.64	30.20	31.63	30.72	29.55	30.38	35.50	0.63	0.246	0.430	0.598
	6 h	64.04^a^	76.38^bc^	93.75^d^	81.33^c^	100.52^d^	68.85^ab^	68.21^a^	63.27^a^	65.07^a^	2.56	<0.001	<0.001	<0.001
	12 h	97.91^cd^	94.29^bcd^	102.91^d^	101.77^d^	129.97^e^	84.12^ab^	88.57^abc^	85.94^ab^	80.8^a^	2.81	<0.001	<0.001	<0.001
	24 h	108.4^ab^	121.79^b^	122.41^b^	120.26^b^	145.76^c^	105.88^ab^	109.77^ab^	100.84^a^	99.62^a^	2.85	<0.001	0.008	<0.001
	48 h	118.61^ab^	141.34^de^	133.77^cde^	144.77^e^	166.87^f^	123.86^abc^	127.87^bcd^	112.32^a^	111.22^a^	3.38	<0.001	0.009	<0.001
A:P	0 h	1.88	1.89	1.87	1.91	1.91	1.96	1.92	1.93	1.92	0.01	0.74	0.20	0.99
	6 h	2.32^e^	1.05^a^	1.08^a^	1.15^b^	1.14^b^	1.40^c^	1.41^c^	1.71^d^	1.75^d^	0.08	<0.001	<0.001	<0.001
	12 h	2.43^e^	1.07^a^	1.08^a^	1.15^b^	1.09^a^	1.44^c^	1.46^c^	1.71^d^	1.71^d^	0.08	<0.001	<0.001	<0.001
	24 h	2.41^e^	1.11^a^	1.11^a^	1.18^a^	1.12^a^	1.49^b^	1.51^b^	1.67^c^	1.70^c^	0.08	<0.001	<0.001	<0.001
	48 h	2.33^e^	1.13^a^	1.13^a^	1.17^a^	1.19^a^	1.53^b^	1.51^b^	1.73^c^	1.71^c^	0.07	<0.001	<0.001	<0.001

^a,b,c,d,e^Different superscripts in the same row imply that their mean values are significantly different (*p* ≤ 0.05). Where: ctrl = Diet 2 (17% hemicellulose); L = diet 2 with low yeast culture; H = diet 2 with high yeast culture; l = diet 2 with low oxalic acid; h = diet 2 with high oxalic acid; Ll = diet 2 with low yeast culture and low oxalic acid; Lh = diet 2 with low yeast culture and high oxalic acid; Hl = diet 2 with high yeast culture and low oxalic acid; Hh = diet 2 with high yeast culture and high oxalic acid; VFA = volatile fatty acid; TVFA = total volatile fatty acid; A:P = acetic acid to propionic acid ratio.

All supplemented treatments in both diets produced higher levels of propionic and total volatile fatty acids compared to the control (*p* < 0.001). The acetic to propionic acid ratio was higher in the control treatments (*p* < 0.001). These results suggest that supplementation with YC and OA may have a buffering effect on the rumen, leading to improved NH_3_-N concentration and volatile fatty acids production.

### 3.3 Rumen microbiome composition

#### 3.3.1 Ruminal bacterial alpha diversity indices

**Figure 3:**
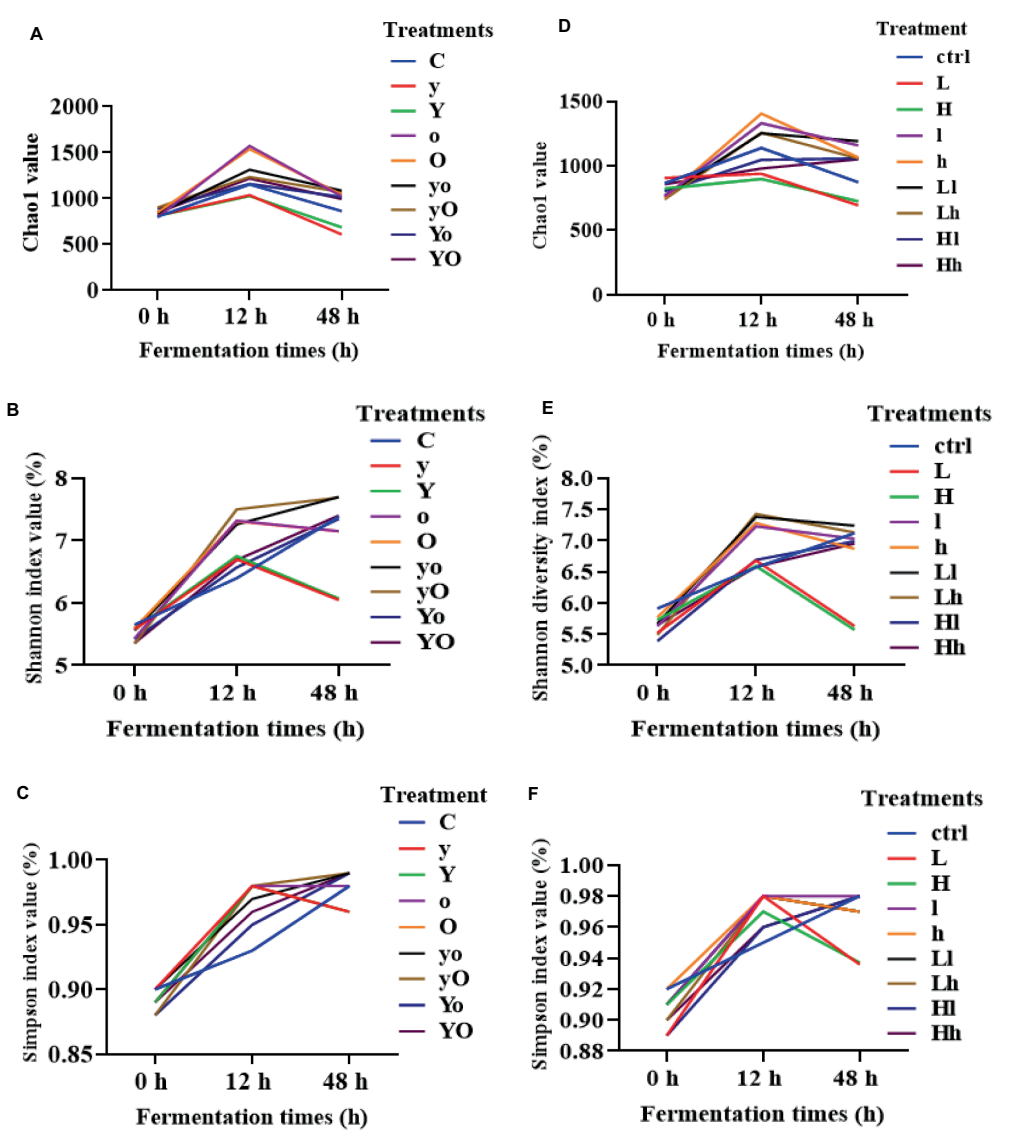
Alpha diversity indices of bacteria community composition of diet 1 [Chao1 **(A)**, Shannon **(B)**, Simpson **(C)**, and diet 2 **(D–F)**] respectively. Where: C = Diet 1 (10.3% hemicellulose); y = diet 1 with low yeast culture; Y = diet 1 with high yeast culture; o = diet 1 with low oxalic acid; O = diet 1 with high oxalic acid; yo = diet 1 with low yeast culture and low oxalic acid; yO = diet 1 with low yeast culture and high oxalic acid; Yo = diet 1 with high yeast culture and low oxalic acid; YO = diet 1 with high yeast culture and high oxalic acid; ctrl = Diet 2 (17% hemicellulose); L = diet 2 with low yeast culture; H = diet 2 with high yeast culture; l = diet 2 with low oxalic acid; h = diet 2 with high oxalic acid; Ll = diet 2 with low yeast culture and low oxalic acid; Lh = diet 2 with low yeast culture and high oxalic acid; Hl = diet 2 with high yeast culture and low oxalic acid; Hh = diet 2 with high yeast culture and high oxalic acid.

**Figure 4:**
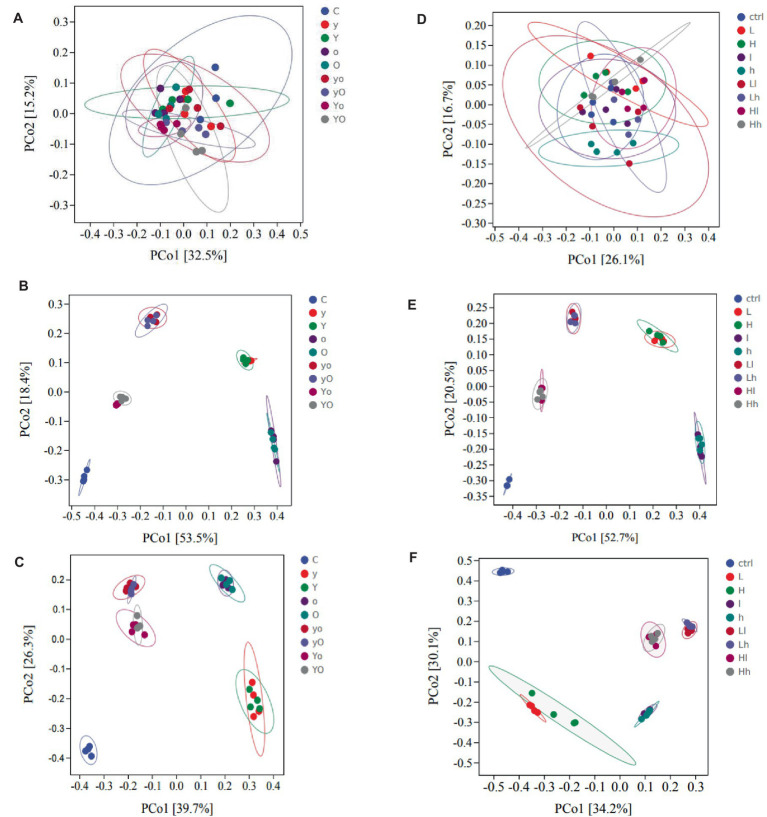
Principal coordinate analysis (PCoA) showing the distribution of the bacterial community composition at the genus level of diet 1 (**A**, 0 h), (**B**, 12 h), and (**C**, 48 h); diet 2 (**D**, 0 h), (**E**, 12 h), and (**F**, 48 h) respectively. Where: C = Diet 1 (10.3% hemicellulose); y = diet 1 with low yeast culture; Y = diet 1 with high yeast culture; o = diet 1 with low oxalic acid; O = diet 1 with high oxalic acid; yo = diet 1 with low yeast culture and low oxalic acid; yO = diet 1 with low yeast culture and high oxalic acid; Yo = diet 1 with high yeast culture and low oxalic acid; YO = diet 1 with high yeast culture and high oxalic acid; ctrl = Diet 2 (17% hemicellulose); L = diet 2 with low yeast culture; H = diet 2 with high yeast culture; l = diet 2 with low oxalic acid; h = diet 2 with high oxalic acid; Ll = diet 2 with low yeast culture and low oxalic acid; Lh = diet 2 with low yeast culture and high oxalic acid; Hl = diet 2 with high yeast culture and low oxalic acid; and Hh = diet 2 with high yeast culture and high oxalic acid. The number of samples included were 108 in each diet. Each point in the plot represents a sample, and different colored dots indicate different treatments. The percentages in square brackets of the axis represent the proportion of sample variance data (distance matrix) that can be interpreted by the corresponding axis. The closer the projection distance of the two points on the axis, the more similar the community composition of the two samples in the corresponding dimension.

For rumen bacteria community composition analyses, a total of 216 samples of ruminal fluid were used, with four replications per fermentation time. In diet 1, supplementation with YC, OA, and their interactions significantly increased the Chao1 value, an indicator of bacterial richness (Figure 3A). Specifically, the OA (*p* < 0.001, at 12 h) and YC and OA interaction (*p* < 0.05, at 48 h) increased the Chao1 values. YC, OA, and their interactions also increased the Shannon (Figure 3B) and Simpson (Figure 3C) diversity indices (*p* < 0.001) at 12 h. In diet 2, the Chao1 index results showed that YC and OA supplementation increased bacterial richness and abundance (Figure 3D). The YC and OA interactions (*p* < 0.001) showed a higher Chao1 index value. However, the Chao1 index value of the YC alone supplemented treatments was significantly lower than that of the control. Supplementation with YC and OA interaction also altered the Shannon (Figure 3E) and Simpson (Figure 3F) diversity indices in this diet. Lower YC and lower OA supplementation significantly increased the Shannon and Simpson indices at 12 h. Additional results on bacterial alpha diversity indices are also shown in Supplementary Figures 1, 2 for diets 1 and 2, respectively. The β-diversity principal coordinate analysis (PCoA) also showed that in each type of supplement, the bacterial community composition was closer to each other at 12 and 48 h but scattered at 0 h in both diets (Figure 4). This PCoA result showed that there is a community composition variation between the control and the YC, OA, and their interaction-supplemented treatments in both diets. However, there is no significant community variation between the lower and higher levels of each supplement. The YC, OA, and their interaction-supplemented treatments bacterial community composition were closer to each other without a significant difference caused by the level of YC and OA.

#### 3.3.2 Ruminal bacterial phylum composition

**Figure 5:**
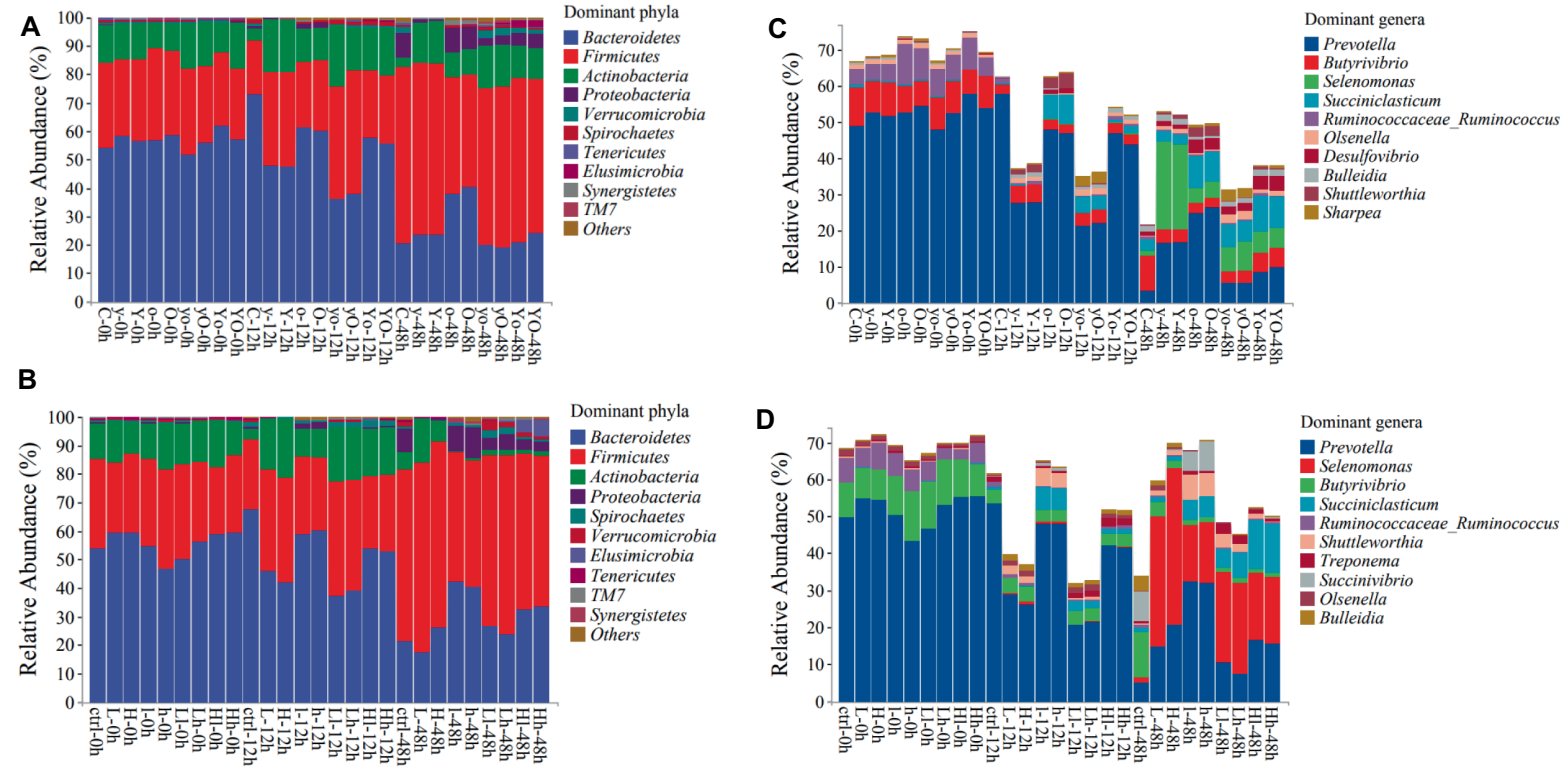
The top 10 dominant bacterial phyla and genera in both diets at different fermentation times [Diet 1 phyla **(A)**, Diet 2 phyla **(B)**, Diet 1 genera **(C)**, and diet 2 genera **(D)**]. Where: C = Diet 1 (10.3% hemicellulose); y = diet 1 with low yeast culture; Y = diet 1 with high yeast culture; o = diet 1 with low oxalic acid; O = diet 1 with high oxalic acid; yo = diet 1 with low yeast culture and low oxalic acid; yO = diet 1 with low yeast culture and high oxalic acid; Yo = diet 1 with high yeast culture and low oxalic acid; YO = diet 1 with high yeast culture and high oxalic acid; ctrl = Diet 2 (17% hemicellulose); L = diet 2 with low yeast culture; H = diet 2 with high yeast culture; l = diet 2 with low oxalic acid; h = diet 2 with high oxalic acid; Ll = diet 2 with low yeast culture and low oxalic acid; Lh = diet 2 with low yeast culture and high oxalic acid; Hl = diet 2 with high yeast culture and low oxalic acid; Hh = diet 2 with high yeast culture and high oxalic acid. In each diet 108 samples were used for analysis.

*Bacteroidetes*, *Firmicutes*, and *Actinobacteria* were the most dominant phyla in both diets. As shown in Table 5 and Figure 5A, in diet 1, supplementation with OA affected the composition and abundance of rumen bacterial phyla. The abundance of *Bacteroidetes* was significantly higher in the control treatment at 12 h. However, at 48 h, OA supplementation significantly increased the abundance of *Bacteroidetes* (*p* < 0.001). Neither YC supplementation alone nor YC and OA interaction had an impact on the abundance of *Firmicutes*. However, their abundance was reduced in treatments supplemented with OA (*p* < 0.001). The YC and OA interactions increased *Actinobacteria* phylum abundance *(p < 0.001)*. The addition of lower levels of YC and OA significantly increased the abundance of the *Spirochaetes* and *Verrucomicrobia* phyla.

**Table 5:** Effects of supplementation with YC and OA in diet 1 on the dominant bacterial phylum composition (%).

Phylum	Time	Diet 1	Yeast culture (YC)		Oxalic acid (OA)		Interactions				SEM	*p-*value		
		C	y	Y	o	O	yo	yO	Yo	YO		YC	OA	YC × OA
*Bacteroidetes*	0 h	54.37	58.15	56.19	56.83	58.68	52.08	55.93	61.67	57.51	0.81	0.29	0.84	0.13
	12 h	72.82^e^	47.71^b^	47.19^b^	61.39^d^	59.88^cd^	36.20^a^	38.10^a^	57.85^cd^	55.41^c^	1.91	<0.001	<0.001	<0.001
	48 h	20.43^a^	23.49^a^	23.69^a^	38.17^b^	40.13^b^	19.88^a^	18.10^a^	20.86^a^	23.91^a^	1.35	<0.001	0.001	<0.001
*Firmicutes*	0 h	29.91	27.12	29.04	32.36	29.69	30.04	26.78	26.27	24.61	0.62	0.02	0.19	0.44
	12 h	18.99^a^	32.94^c^	33.66^cd^	23.05^ab^	25.39^b^	39.55^de^	43.46^e^	23.79^ab^	24.21^ab^	1.38	<0.001	0.048	<0.001
	48 h	61.85^b^	61.00^b^	60.24^b^	40.57^a^	39.83^a^	55.62^b^	56.67^b^	58.07^b^	54.51^b^	1.41	<0.001	<0.001	<0.001
*Actinobacteria*	0 h	13.18	12.44	12.45	9.03	9.39	15.71	15.76	10.38	16.27	0.75	0.07	0.47	0.18
	12 h	3.92^a^	19.07^cd^	18.66^cd^	11.67^b^	11.08^b^	21.41^d^	14.71^bc^	14.88^bc^	16.85^bcd^	0.93	<0.001	0.13	<0.001
	48 h	3.73^a^	13.59^cd^	14.51^d^	9.33^b^	8.84^b^	14.478^d^	15.07^d^	11.16^bc^	10.98^bc^	0.61	<0.001	0.10	<0.001
*Proteobacteria*	0 h	0.11	0.09	0.10	0.11	0.13	0.06	0.07	0.06	0.07	0.01	0.02	0.16	0.79
	12 h	0.98^b^	0.06^a^	0.10^a^	1.82^c^	1.87^c^	0.20^a^	0.23^a^	0.10^a^	0.09^a^	0.12	<0.001	<0.001	<0.001
	48 h	8.43^c^	1.43^a^	1.17^a^	8.45^c^	7.97^c^	2.91^ab^	2.84^ab^	4.40^b^	4.67^b^	0.50	<0.001	0.003	0.021
*Spirochaetes*	0 h	0.95^b^	0.80^ab^	0.67^ab^	0.44^a^	0.55^ab^	0.70^ab^	0.54^ab^	0.46^a^	0.52^a^	0.04	0.19	<0.001	0.19
	12 h	1.39^cd^	0.03^a^	0.07^a^	0.80^b^	0.74^b^	1.00^bc^	1.36^cd^	1.57^d^	1.77^d^	0.11	0.04	<0.001	<0.001
	48 h	0.49^bc^	0.05^a^	0.05^a^	0.80^cde^	0.72^bcd^	1.12^e^	1.04^de^	0.40^ab^	0.68^bcd^	0.07	<0.001	<0.001	<0.001
*Verrucomicrobia*	0 h	0.73^bc^	0.63^abc^	0.79^c^	0.49^abc^	0.76^c^	0.67^abc^	0.36^a^	0.54^abc^	0.39^ab^	0.03	0.17	0.003	0.003
	12 h	1.22^d^	0.00^a^	0.00^a^	0.00^a^	0.00^a^	0.56^bc^	0.72^c^	0.47^bc^	0.43^b^	0.07	0.02	0.34	<0.001
	48 h	2.55^bc^	0.004^a^	0.01^a^	0.02^a^	0.03^a^	2.84^c^	2.88^c^	1.82^b^	1.79^b^	0.21	<0.001	<0.001	<0.001
*Elusimicrobia*	0 h	0.00	0.00	0.00	0.00	0.00	0.00	0.00	0.00	0.00	0.00	0.45	0.45	0.122
	12 h	0.00^a^	0.00^a^	0.01^a^	0.06^a^	0.06^a^	0.02^a^	0.02^a^	0.61^c^	0.49^b^	0.04	<0.001	<0.001	<0.001
	48 h	0.00^a^	0.02^a^	0.02^a^	0.16^a^	0.14^a^	0.11^a^	0.11^a^	2.44^b^	2.26^b^	0.09	<0.001	<0.001	<0.001

^a,b,c,d,e^Different superscripts in the same row imply that their mean values are significantly different (*p* ≤ 0.05). Where: C = Diet 1 (10.3% hemicellulose); y = diet 1 with low yeast culture; Y = diet 1 with high yeast culture; o = diet 1 with low oxalic acid; O = diet 1 with high oxalic acid; yo = diet 1 with low yeast culture and low oxalic acid; yO = diet 1 with low yeast culture and high oxalic acid; Yo = diet 1 with high yeast culture and low oxalic acid; and YO = diet 1 with high yeast culture and high oxalic acid.

Adding YC and OA to diet 2 also increased the number of *Bacteroidetes*, *Firmicutes*, *Actinobacteria*, and *Proteobacteria*, which are the most dominant phyla (*p* < 0.001; Table 6; Figure 5B). Specifically, treatments supplemented with OA alone showed a higher relative abundance of the *Bacteroidetes* phylum (*p* < 0.001). The lower levels of YC and OA supplementation significantly increased the abundance of the *Bacteroidetes* phylum (*p* < 0.001). The relative abundance of *Firmicutes* was also significantly increased with the addition of YC at both higher and lower levels (*p* < 0.001).

**Table 6:** Effects of YC and OA supplementation in diet 2 on the dominant bacterial phylum composition (%).

Phylum	Time	Diet 2	Yeast culture (YC)		Oxalic acid (OA)		Interactions				SEM	*p* value		
		ctrl	L	H	l	h	Ll	Lh	Hl	Hh		YC	OA	YC × OA
*Bacteroidetes*	0 h	53.89^abc^	59.20^c^	59.18^c^	54.51^abc^	46.87^a^	50.16^ab^	56.08^bc^	58.70^c^	59.48^c^	0.89	<0.001	0.063	0.006
	12 h	67.87^e^	45.73^b^	41.83^ab^	58.99^d^	60.14^d^	37.17^a^	38.87^a^	53.64^c^	52.88^c^	1.71	<0.001	0.112	<0.001
	48 h	21.33^ab^	17.41^a^	26.13^bc^	42.38^d^	40.65^d^	26.59^bc^	23.93^ab^	32.71^c^	33.33^c^	1.43	<0.001	<0.001	<0.001
*Firmicutes*	0 h	31.12^ab^	24.42a	27.97^ab^	30.53^ab^	34.80^b^	33.21^ab^	28.21^ab^	23.52^a^	27.26^ab^	0.87	0.009	0.45	0.04
	12 h	24.15^a^	35.91^b^	36.89^b^	27.37^a^	25.58^a^	40.14^b^	39.14^b^	25.63^a^	27.00^a^	1.08	<0.001	0.115	<0.001
	48 h	60.35^cd^	66.22^d^	65.00^d^	44.94^a^	44.27^a^	60.16^bcd^	62.82^d^	54.40^bc^	52.83^b^	1.38	<0.001	<0.001	0.006
*Actinobacteria*	0 h	12.85	14.78	10.94	12.87	16.08	14.51	14.21	16.35	11.51	0.64	0.612	0.554	0.282
	12 h	4.06^a^	17.86^cd^	20.90^d^	9.30^b^	10.22^b^	19.27^cd^	18.17^cd^	16.64^c^	16.60^c^	0.92	<0.001	0.331	<0.001
	48 h	5.95^a^	15.69^b^	7.50^a^	0.69^a^	0.85^a^	1.64^a^	1.85^a^	1.53^a^	1.67^a^	0.91	0.014	<0.001	0.026
*Proteobacteria*	0 h	0.16^ab^	0.13^ab^	0.15^ab^	0.17^b^	0.11^ab^	0.12^ab^	0.09^ab^	0.05^a^	0.07^ab^	0.01	0.028	0.023	0.156
	12 h	0.51^b^	0.11^a^	0.11^a^	2.07^c^	2.13^c^	0.17^a^	0.24^a^	0.19^a^	0.18^a^	0.13	<0.001	<0.001	<0.001
	48 h	8.43^de^	0.39^a^	1.08^ab^	8.76^de^	10.47^e^	4.34^bc^	5.48^cd^	3.46^abc^	3.20^abc^	0.60	<0.001	<0.001	0.105
*Spirochaetes*	0 h	0.46^ab^	0.56^b^	0.47^ab^	0.48^ab^	0.44^ab^	0.40^ab^	0.24^a^	0.40^ab^	0.41^ab^	0.02	0.511	0.039	0.105
	12 h	1.38^cde^	0.11^a^	0.05^a^	0.86^bc^	0.68^ab^	1.20^bcd^	1.77^def^	2.40^f^	1.86^ef^	0.13	<0.001	<0.001	<0.001
	48 h	0.77^b^	0.03^a^	0.04^a^	1.03^b^	0.97^b^	2.88^c^	2.34^c^	1.16^b^	0.99^b^	0.16	<0.001	<0.001	<0.001
*Verrucomicrobia*	0 h	0.58	0.43	0.56	0.60	0.65	0.69	0.37	0.38	0.56	0.03	0.245	0.893	0.074
	12 h	1.33^c^	0.01^a^	0.01^a^	0.01^a^	0.003^a^	0.65^b^	0.56^b^	0.35^ab^	0.37^ab^	0.08	0.022	0.147	<0.001
	48 h	1.11^b^	0.01^a^	0.01^a^	0.001^a^	0.01^a^	2.82^d^	1.90^c^	1.19^b^	1.28^b^	0.17	<0.001	<0.001	<0.001

^a,b,c,d,e,f^Different superscripts in the same row imply that their mean values are significantly different (*p* ≤ 0.05). Where: ctrl = Diet 2 (17% hemicellulose); L = diet 2 with low yeast culture; H = diet 2 with high yeast culture; l = diet 2 with low oxalic acid; h = diet 2 with high oxalic acid; Ll = diet 2 with low yeast culture and low oxalic acid; Lh = diet 2 with low yeast culture and high oxalic acid; Hl = diet 2 with high yeast culture and low oxalic acid; and Hh = diet 2 with high yeast culture and high oxalic acid.

#### 3.3.3 Rumen bacterial genus composition

*Prevotella, Selenomonas, Butyrivibrio, Succiniclasticum*, and *Ruminococcaceae_Ruminococcus* were the dominant genera identified in both diets. The results of the bacterial genus composition in diet 1 are shown in Table 7; Figure 5C, and Supplementary Table 1. Hence, YC, OA, and their interaction increased the *Prevotella* genus abundance at 48 h (*p* < 0.001). The supplemented treatments also showed a significantly higher abundance of the S*elenomonas* genus at 48 h. However, the *Butyrivibiro* genus was higher in the control treatment. In diet 2, the *Prevotella* genus was found to have a higher abundance in the supplemented experimental treatments than in the control (*p* < 0.001; Table 8; Figure 5D; Supplementary Table 2). S*elenomonas* and *Succiniclasticum* were also the dominant genera, which were highly increased in the supplemented treatments (*p* < 0.001). The *Butyrivibiro* genus was higher in the control treatment. However, this genus was significantly increased in higher YC supplemented treatment at 12 h. As shown in Figure 5, the dominant bacterial genera were highly distributed in the supplemented treatments.

**Table 7:** Effects of YC and OA supplementation in diet 1 on ruminal bacterial genus composition (%).

Genus	Time	Diet 1	Yeast culture (YC)		Oxalic acid (OA)		Interactions				SEM	*p* value		
		C	y	Y	o	O	yo	yO	Yo	YO		YC	OA	YC × OA
*Prevotella*	0 h	49.12	52.77	51.66	52.79	54.50	47.85	52.33	57.84	53.71	0.88	0.265	0.523	0.298
	12 h	57.99^e^	27.53^bc^	27.98^c^	48.03^d^	46.75^d^	21.40^a^	21.83^ab^	46.99^d^	43.69^d^	2.17	<0.001	0.386	<0.001
	48 h	3.43^a^	16.52^c^	16.92^c^	24.89^d^	26.45^d^	5.27^ab^	5.37^ab^	8.61^b^	9.99^b^	1.40	<0.001	0.159	<0.001
*Selenomonas*	0 h	0.01^ab^	0.02^ab^	0.02^ab^	0.03^b^	0.02^ab^	0.01^ab^	0.01^ab^	0.00^a^	0.01^ab^	0.002	0.067	0.212	0.016
	12 h	0.00^a^	0.21^b^	0.15^ab^	0.48^c^	0.48^c^	0.10^ab^	0.16^ab^	0.08^ab^	0.06^ab^	0.03	<0.001	0.002	<0.001
	48 h	1.32^a^	24.55^c^	23.53^c^	4.18^ab^	4.29^ab^	6.98^ab^	7.94^b^	5.96^ab^	5.42^ab^	1.41	<0.001	<0.001	<0.001
*Butyrivibrio*	0 h	10.66^b^	8.64^ab^	9.30^ab^	7.17^ab^	6.92^a^	9.05^ab^	9.14^ab^	6.67^a^	8.97^ab^	0.31	0.495	0.021	0.035
	12 h	2.40^a^	4.65^d^	4.64^d^	2.68^ab^	2.41^a^	3.38^bc^	3.92^cd^	2.79^ab^	2.99^ab^	0.15	<0.001	<0.001	<0.001
	48 h	9.70^c^	3.57^ab^	3.48^ab^	2.48^a^	2.58^a^	3.10^a^	3.54^ab^	5.15^b^	5.06^b^	0.37	<0.001	<0.001	<0.001
*Succiniclasticum*	0 h	0.45^ab^	0.39^ab^	0.58^b^	0.23^ab^	0.30^ab^	0.38^ab^	0.29^ab^	0.14^ab^	0.12^a^	0.03	0.488	0.001	0.064
	12 h	0.47^ab^	0.15^a^	0.04^a^	6.26^e^	7.92^e^	4.20^d^	3.52^cd^	0.94^ab^	2.16^bc^	0.47	<0.001	<0.001	<0.001
	48 h	3.01^a^	2.79^a^	2.65^a^	9.20^c^	8.52^c^	6.41^b^	5.81^b^	9.98^c^	8.81^c^	0.48	<0.001	<0.001	<0.001
*Ruminococcaceae_Ruminococcus*	0 h	4.78^a^	4.50^a^	4.86^a^	1.17^b^	8.75^ab^	7.64^ab^	6.98^ab^	8.95^ab^	5.25^a^	0.53	0.086	<0.001	0.412
	12 h	1.12^e^	0.51^bc^	0.76^cd^	0.19^a^	0.17^a^	0.35^ab^	0.43^ab^	0.94^de^	0.72^cd^	0.06	<0.001	<0.001	<0.001
	48 h	0.65^c^	0.35^ab^	0.33^ab^	0.30^a^	0.29^a^	0.38^ab^	0.37^ab^	0.50^bc^	0.45^ab^	0.02	0.117	0.073	<0.001
*Shuttleworthia*	0 h	0.11^ab^	0.11^ab^	0.20^b^	0.21^b^	0.21^b^	0.10^ab^	0.05^a^	0.11^ab^	0.04^a^	0.01	0.004	0.212	0.006
	12 h	0.01^a^	1.41^b^	2.20^c^	3.11^d^	4.00^e^	0.30^a^	0.40^a^	0.02^a^	0.02^a^	0.24	<0.001	0.004	<0.001
	48 h	0.21^a^	0.75^a^	0.79^a^	2.91^b^	2.81^b^	0.32^a^	0.55^a^	0.42^a^	0.37^a^	0.18	<0.001	<0.001	<0.001

^a,b,c,d,e^Different superscripts in the same row imply that their mean values are significantly different (*p* ≤ 0.05). Where: C = Diet 1 (10.3% hemicellulose); y = diet 1 with low yeast culture; Y = Diet 1 with high yeast culture; o = diet 1 with low oxalic acid; O = diet 1 with high oxalic acid; yo = diet 1 with low yeast culture and low oxalic acid; yO = diet 1 with low yeast culture and high oxalic acid; Yo = diet 1 with high yeast culture and low oxalic acid; and YO = diet 1 with high yeast culture and high oxalic acid.

**Table 8:** Effects of supplementation with YC and OA in diet 2 on the dominant genus composition of ruminal bacteria (%).

Genus	Time	Diet 2	Yeast culture (YC)		Oxalic acid (OA)		Interactions				SEM	*p* value		
		ctrl	L	H	l	h	Ll	Lh	Hl	Hh		YC	OA	YC × OA
*Prevotella*	0 h	49.77^ab^	54.89^b^	54.37^b^	50.53^ab^	43.24^a^	46.56^ab^	53.10^b^	55.23^b^	55.47^b^	0.88	<0.001	0.239	0.01
	12 h	53.47^e^	29.00^b^	26.31^b^	47.91^d^	48.13^d^	20.57^a^	21.47^a^	42.10^c^	41.65^c^	2.01	<0.001	0.542	<0.001
	48 h	4.84^a^	14.70^bc^	20.84^d^	32.20^e^	32.12^e^	10.43^ab^	7.30^a^	16.73^cd^	15.55^bcd^	1.60	<0.001	<0.001	<0.001
*Selenomonas*	0 h	0.00^a^	0.02^ab^	0.01^ab^	0.02^b^	0.022^b^	0.02^b^	0.02^b^	0.02^b^	0.02^b^	0.00	1.99	<0.001	2.228
	12 h	0.02^a^	0.35^bcd^	0.37^cd^	0.73^e^	0.56^de^	0.16^abc^	0.09^a^	0.13^ab^	0.07^a^	0.04	<0.001	0.021	<0.001
	48 h	1.58^a^	35.52^cd^	42.18^d^	15.37^ab^	16.17^b^	24.75^bc^	24.70^bc^	17.91^b^	18.03^b^	2.08	<0.001	0.009	<0.001
*Butyrivibrio*	0 h	9.54^ab^	8.11^a^	8.26^a^	10.39^abc^	13.68^d^	12.85^cd^	12.22^bcd^	10.30^abc^	8.59^a^	0.38	<0.001	<0.001	0.002
	12 h	3.61^abc^	3.93^bc^	4.40^c^	3.20^ab^	3.00^a^	3.78^abc^	3.62^abc^	3.23^ab^	3.63^abc^	0.09	0.004	<0.001	0.106
	48 h	12.17^c^	3.61^b^	2.04^a^	1.35^a^	1.52^a^	0.85^a^	1.18^a^	1.20^a^	1.24^a^	0.58	<0.001	<0.001	<0.001
*Succiniclasticum*	0 h	0.14	0.23	0.14	0.11	0.13	0.18	0.23	0.07	0.22	0.02	0.099	0.203	0.717
	12 h	0.94^ab^	0.07^a^	0.07^a^	6.10^c^	5.85^c^	2.50^b^	1.92^ab^	1.17^ab^	1.47^ab^	0.38	<0.001	<0.001	<0.001
	48 h	1.57^a^	1.42^a^	1.25^a^	5.48^b^	5.66^b^	5.31^b^	7.07^b^	13.09^c^	13.04^c^	0.74	<0.001	<0.001	<0.001
*Ruminococcaceae_Ruminococcus*	0 h	6.46	5.45	7.24	6.07	5.77	5.31	3.01	2.72	5.44	0.46	0.396	0.239	0.332
	12 h	1.23^c^	0.86^b^	0.80^b^	0.26^a^	0.26^a^	0.51^a^	0.46^a^	0.82^b^	0.88^b^	0.05	<0.001	<0.001	<0.001
	48 h	0.55^d^	0.26^abc^	0.27^bc^	0.22^ab^	0.22^ab^	0.13^a^	0.21^ab^	0.36^c^	0.33^bc^	0.02	<0.001	<0.001	<0.001
*Shuttleworthia*	0 h	0.14	0.10	0.13	0.15	0.20	0.25	0.10	0.04	0.18	0.02	0.657	0.785	0.203
	12 h	0.00^a^	2.45^b^	1.82^b^	4.99^d^	4.06^c^	0.49^a^	0.64^a^	0.02^a^	0.02^a^	0.30	<0.001	0.022	<0.001
	48 h	0.29^a^	0.29^a^	1.41^ab^	6.87^d^	5.94^cd^	3.76^bc^	2.03^ab^	1.33^ab^	0.38^a^	0.41	<0.001	<0.001	<0.001

^a,b,c,d,e^Different superscripts in the same row imply that their mean values are significantly different (*p* ≤ 0.05). Where: ctrl = diet 2 (17% hemicellulose); L = diet 2 with low yeast culture; H = diet 2 with high yeast culture; l = diet 2 with low oxalic acid; h = diet 2 with high oxalic acid; Ll = diet 2 with low yeast culture and low oxalic acid; Lh = diet 2 with low yeast culture and high oxalic acid; Hl = diet 2 with high yeast culture and low oxalic acid; and Hh = diet 2 with high yeast culture and high oxalic acid.

### 3.4 The relationship between rumen fermentation parameters and bacterial genus composition

A canonical correlation analysis (CCA) was used to analyze Pearson’s correlation coefficient for multiple sets of variables as shown in Table 9. In diet 1, *Prevotella* and *Selenomonas* showed significant and positive correlations with IVDMD, propionic acid, and TVFA. The *Sicciniclasticum* genus showed significant and positive correlations with IVCPD, pH, acetic acid, butyric acid, and NH_3_-N. However, *Prevotella* showed a significant and negative correlation with NH_3_-N. In diet 2, the *Prevotella* genus showed a positive correlation with IVCPD, pH, acetic acid, propionic acid, and TVFA, but had a negative correlation with IVDMD, IVNDFD, NH_3_-N, butyric acid, and the acetic to propionic acid ratio. The *Butyrivibrio* genus had a positive association with NH_3_-N, acetic acid, butyric acid, and the acetic to propionic acid ratio but showed a negative correlation with other parameters. The *Selenomona*s genus exhibits a significant positive association with pH, propionic acid, butyric acid, TVFA, and the ratio of acetic acid to propionic acid. Conversely, it demonstrated a negative correlation with *in vitro* disappearance measures, NH_3_-N, acetic acid, and propionic acid.

**Table 9:** The correlation between ruminal bacteria community composition (genus) and nutritional disappearance, and rumen fermentation parameters in the two diets.

Item	Genus	Nutritional disappearance and rumen fermentation parameters									
		IVDMD	IVNDFD	IVCPD	pH	NH_3_-N	Acetic	Propionic	Butyric	TVFA	A:P
Diet 1	*Prevotella*	0.43^*^	0.24	−0.02	−0.05	−0.82^**^	−0.30	0.86^**^	−0.43^*^	0.42^*^	−0.75^**^
	*Selenomonas*	0.41^*^	0.07	−0.36	−0.47^*^	0.03	−0.49^*^	0.50^*^	0.22	0.25	−0.56
	*Butyrivibrio*	−0.32	−0.68	−0.02	−0.50^*^	0.22	0.66^**^	−0.71^**^	0.46^*^	−0.12	0.90^**^
	*Succiniclasticum*	−0.52	0.37	0.44^*^	0.66^**^	−0.37	−0.21	−0.12	−0.82^**^	−0.49^*^	−0.06
	*Ruminococcaceae_Ruminococcus*	−0.41	−0.64	0.04	−0.34	0.26	0.56*	−0.71^**^	0.39^*^	−0.20	0.81^**^
	*Shuttleworthia*	0.40	0.24	0.06	0.18	−0.75^**^	−0.12	0.65^**^	−0.52^*^	0.30	−0.53*
	*Olsenella*	−0.21	0.46^*^	−0.04	0.45	0.77^**^	−0.34	−0.23	0.18	−0.29	−0.06
	*Bulleidia*	−0.39	−0.41^*^	−0.10	−0.56^*^	0.40^*^	0.17	−0.44^*^	0.48^*^	−0.13	0.43^*^
	*Treponema*	−0.12	0.21	0.35	0.73^*^	0.29	0.03	−0.32	−0.24	−0.33	0.18
Diet 2	*Prevotella*	−0.19	−0.09	0.13	0.04	−0.78^**^	0.50^*^	0.75^**^	−0.34	0.66^**^	−0.62^**^
	*Selenomonas*	−0.32	−0.12	−0.05	0.10	−0.41^*^	−0.36	0.35	0.09	0.15	−0.63^**^
	*Butyrivibrio*	−0.30	−0.15	−0.57^*^	−0.77^**^	0.34	0.13	−0.39	0.49^*^	−0.18	0.66^**^
	*Succiniclasticum*	0.82^**^	0.27	0.81^**^	0.74^**^	0.24	−0.24	−0.36	−0.77^**^	−0.45^*^	0.20
	*Ruminococcaceae_Ruminococcus*	0.08	−0.15	−0.17	−0.44	0.30	−0.09	−0.57^*^	−0.03	−0.45^*^	0.72^**^
	*Shuttleworthia*	−0.15	0.05	0.11	0.15	−0.34	0.60^**^	0.64^**^	−0.04	0.67^**^	−0.49^**^
	*Olsenella*	−0.56^*^	−0.13	−0.47^*^	−0.41	−0.29	−0.28	0.11	0.38	0.03	−0.19
	*Bulleidia*	−0.38	−0.17	−0.63^**^	−0.78^**^	0.27	0.00	−0.36	0.52^*^	−0.20	0.57^*^
	*Treponema*	0.54^*^	0.25	0.31	0.62^**^	0.69^**^	−0.09	−0.34	−0.05	−0.27	0.23

^**^Indicates that *p* < 0.001, and ^*^indicates when 0.05 ≥ *p* ≥ 0.001. The significance arrangement is columnwise. Where: IVDMD = *in vitro* dry matter disappearance; IVNDFD = *in vitro* neutral detergent fiber disappearance; IVCPD = *in vitro* crude protein disappearance; NH_3_-N = ammonia nitrogen; TVFA = total volatile fatty acid, and A:P = acetic to propionic acid ratio.

## 4 Discussion

### 4.1 *In vitro* nutrient disappearance (IVDMD, IVNDFD, IVCPD)

One of the rumen fermentation efficiency measuring parameters is nutrient digestibility or disappearance. However, the researchers have found inconsistent results regarding the effects of yeast culture and oxalic acid on nutrient digestibility. In this study, YC supplementation in the first experiment (diet 1) increased IVDMD and IVNDFD. Similarly, another report showed that supplementing heifers’ diets with YC increased DM and NDF digestibility (12) by promoting the growth of fiber-digesting bacteria (33). Supplementing the diet with a lower level of OA also increased the IVDMD and IVNDFD. This result partially contradicts the report by Benbati et al. (18), which stated that DM digestibility was reduced due to OA administration in sheep, but returned to normal after 14 days, possibly due to the responsible microbes adapting to the supplement. The interaction of lower YC and lower OA increased the IVCPD. In the second experiment (diet 2), OA and interaction supplementation increased the IVDMD, while YC alone had no effect on IVDMD. Studies have reported that YC has inconsistent effects on hemicellulose digestibility in ruminants. The addition of YC to the diet did not show a significant impact on hemicellulose digestibility (34). However, another study has shown that YC supplementation could increase hemicellulose digestibility (35). The interaction of YC and OA also improved IVCPD, which is consistent with a report that found feeding different levels of YC increased CP digestibility (36). However, another researcher reported that oxalic acid has no significant effect on CP digestibility in goats (37). This result suggests that the addition of YC and OA may improve nutrient disappearance.

### 4.2 Rumen fermentation parameters

#### 4.2.1 Ruminal pH

In ruminant nutrition, the optimal ruminal pH is an indicator of the rumen’s performance and the overall health of the animal (38). In modern profit-driven sheep production systems, it is common for ruminal pH to decrease and for subacute ruminal acidosis (SARA) to occur. Therefore, it is necessary to buffer and maintain an optimal ruminal pH in an intensive sheep production system. In both diets, YC and OA supplementation significantly increased the pH levels at different fermentation times. Similarly, reports have shown that YC might have a buffering effect on the rumen (39, 40). Nevertheless, studies examining the impact of OA have shown that it does not have a statistically significant influence on rumen pH (41). Therefore, supplementing with YC and OA may help to stabilize the rumen environment. This increase in ruminal pH may imply an improvement in fiber digestion, protein degradation, microbial activities, nutrient absorption, and overall rumen health.

#### 4.2.2 Ammonia nitrogen concentration

Ruminants have a special stomach physiology that enables them to utilize different forms of nitrogen as protein sources. Ammonia nitrogen in the rumen is derived from the degradation of dietary protein and nonprotein nitrogen sources. It serves as an indicator of rumen internal performance, being continuously produced in the rumen, and is essential for microbes to synthesize microbial protein (42). In both diets, YC and OA interactions significantly increased NH_3_-N concentrations at different fermentation times. However, supplementing with either YC or OA separately reduced NH_3_-N concentrations in both diets. Similarly, Habeeb (43) found that YC supplements could lead to reduced NH_3_-N. On the other hand, in lambs fed orchard grass, the addition of YC did not affect rumen NH_3_-N (44). Studies have reported that YC supplementation reduces NH_3_-N concentration in the rumen (11, 12, 45). This may be because YC supplementation depresses ammonia-producing microbes (46). The current finding, however, suggests that combining YC and OA supplements could have a positive effect on increasing NH_3_-N levels, ultimately leading to an increase in microbial protein synthesis in the rumen. Moreover, supplementing YC and OA together might reduce the inhibitory effects of OA on NH_3_-N in the rumen, which could be due to the deamination and proteolysis effects of OA (41). The higher ammonia nitrogen concentration resulted in increased microbial protein synthesis, improved nitrogen utilization, and enhanced dietary protein degradation.

#### 4.2.3 Volatile fatty acids production

Volatile fatty acids are the main energy sources and precursors for the production of animal products. They are produced through the bioconversion of diets in the rumen by anaerobic digestion (47). In both diets, supplementation with OA at lower and higher levels as well as the interaction between YC and OA significantly increased the production of acetic acid. This suggests that OA supplementation alone had a greater impact on acetic acid production than YC and their interactions. This indicates that OA may facilitate the degradation of fiber and increase nutrient availability for the host. However, at 24 and 48 h, the control produced more acetic acid. Supplementation with YC and OA showed significantly higher TVFA production compared to the control. Individual supplementation of YC and OA had a greater impact on TVFA production than their interactions. Although there is a lack of reports on the effects of OA, many researchers have reported that YC supplementation has positive effects on increasing acetic, propionic, and butyric acids (43, 48). Therefore, the combination of YC and OA might have a promising effect on boosting essential volatile fatty acid production. Furthermore, YC and OA supplementation significantly increased propionic acid production in both diets. However, the acetic acid to propionic acid ratio was significantly lower in the supplemented treatments in both diets. A study reported that YC supplementation could significantly increase propionic and TVFA production, but reduce acetic acid, resulting in a lower acetic to propionic acid ratio (49). Hence, YC and OA supplementation may enhance VFA production in the rumen by improving rumen functions. Increased volatile fatty acid production may lead to improved energy yield, enhanced rumen efficiency, better nutrient absorption, maintained pH levels, efficient fiber digestion, and increased microbial activities.

### 4.3 Rumen bacterial community composition

#### 4.3.1 Bacterial species diversity

The rumen ecosystem contains diverse microbes, predominantly bacteria (50). Factors that affect the diversity of bacterial species include diet, dietary supplements, host species, and environmental factors (51). Species diversity can be measured using different alpha diversity indices, such as the Chao1 index and the Shannon and Simpson diversity indices, which quantify the richness of species in a community (52). In diet 1, the addition of OA alone, as well as the interaction of YC and OA, increased species diversity. This aligns with a previous study that found an increase in the richness and diversity of rumen microbiota when organic acids were added to high-grain diets (53). However, this partially contradicts an other study that found oxalic acid reduced the microbial diversity in sheep, but the diversity gradually returned to normal (41). YC and OA supplementation also affected the Shannon and Simpson diversity indices of the bacterial community. Treatments supplemented with YC and OA interactions showed significantly higher Shannon diversity index values. In diet 2, the YC and OA interactions significantly increased the Chao1 index values, indicating higher bacterial species richness. However, YC alone showed significantly lower Chao1 values. Supplementing YC and OA together significantly increased the Shannon and Simpson indices. This implies that YC and OA may have a synergistic effect as a supplement to enhance rumen bacterial richness and diversity, contributing to the overall stability and resilience of the rumen ecosystem. A diverse bacterial community can help the animal cope with different stresses and disturbances by maintaining various functions in the body. Furthermore, diverse bacterial communities may help the animal withstand different diseases and environmental impacts.

#### 4.3.2 Rumen bacterial phylum composition

The composition of ruminal bacterial phyla varies across different parts of the rumen, however, *Bacteroidetes* and *Firmicutes* are the most dominant phyla (54). This result showed that *Bacteroidetes* comprised 45.06%, *Firmicutes* 37.39%, and *Actinobacteria* 11.70% of the total bacterial composition. Similarly, a study found that *Bacteroidetes* (40.95%) and *Firmicutes* (36.36%) are dominant phyla in sheep rumen (55). The main fiber fermenters such as *Prevotella*, *Butyrivibrio*, and *Pseudobutyrivibrio* are members of the phyla *Bacteroidetes* and *Firmicutes* (56), which are also efficient hemicellulose fermenters (50). In the first experiment (diet 1), the relative abundance of *Bacteroidetes* was higher in the control at 12 h; however, at 48 h, OA supplementation significantly increased the abundance, possibly due to the bacteria acclimatizing to oxalic acid. The abundance of *Firmicutes* was not affected. However, supplementation with OA reduced the relative abundance of *Firmicutes*. The interaction between YC and OA increased the abundance of the *Actinobacteria* phylum, which contains the crucial probiotic *Bifidobacterium* genus for gut health (7). Additionally, supplementation with YC and OA interaction significantly increased the abundance of the *Spirochaetes* and *Verrucomicrobia* phyla. The phyla *Spirochaetes* and *Verrucomicrobia* have been identified as possible producers and facilitators of enzymes involved in the degradation of complex polysaccharides such as xyloglucans, peptidoglycans, and pectin (57). In the second experiment (diet 2), supplementation of OA increased the abundance of the *Bacteroidetes* phylum. The YC and OA interaction also significantly increased the composition of this phylum. Both low and high levels of YC supplementation increased the abundance of *Firmicutes*. Furthermore, lower level of YC and higher level of OA, and lower levels of both YC and OA supplementation, also increased the abundance of the *Firmicutes* phylum. The supplementation of YC and OA also increased abundance of the *Actinobacteria* phylum. These results indicate that the interaction of YC and OA enhanced the composition of the rumen bacterial community at the phylum level. Overall, these findings suggest that the supplementation of YC and OA may have a promising effect on increasing the abundance of beneficial bacterial phyla which are primary fermenters of cellulose and hemicellulose (50, 56).

#### 4.3.3 Rumen bacterial genera composition

The dominant genera identified in the recent *in vitro* experiment were *Prevotella, Selenomonas, Butyrivibrio, Succiniclasticum, Ruminococcaceae_Ruminococcus, Shuttleworthia, and Olsenella*. In both diets, YC and OA supplemented treatments increased the abundance of the *Prevotella* genus. Individual supplementation with YC and OA also significantly increased the abundance of this genus. The *Selenomonas* and *Succiniclasticum* genera were dominant and showed a significant increase in the supplemented treatments. However, the *Butyrivibrio* genus was higher in the controls than in the supplemented treatments at 12 and 48 h in both diets. *Prevotella* strains have been found to ferment hemicellulose but exhibit differences in fermentation end products (58). It contains the *Prevotella ruminicola* species which plays a role in nitrogen metabolism and is also a fibrolytic bacteria that possesses carbohydrate esterases, facilitating hemicellulose degradation (59). The *Butyrivibrio* genus can also degrade hemicellulose (arabinoxylans) and pectin and produce butyrate (60). In both diets, the supplementation also showed a higher *Selenomonas* genus at 48 h. It contains the *Selenomonas bovis* species, which could ferment various sugars and produce acetate, propionate, and CO_2_ as end products (61, 62). Studies have reported that *Selenomonas* enhances the proliferation of the microbial community in the rumen (63). Additionally, it encompasses several species that actively participate in the process of nitrate and nitrite reduction inside the rumen (64) and could facilitate propionic acid producing bacteria (65).

This *in vitro* experiment showed that adding YC and OA to hemicellulosic diets significantly altered the composition of beneficial rumen bacterial genera. This could be attributed to the creation of a favorable environment for bacterial growth and performance by the supplemented ingredients. As the pH results indicated, YC and OA supplementation increased the pH level of the ruminal fluid. Additionally, the ammonia nitrogen concentration increased in these experiments, suggesting that bacteria were able to obtain sufficient microbial protein, thereby facilitating their proliferation in the rumen. Hence, this may explain the significant increase in rumen bacteria at the genus and phylum taxonomic levels.

#### 4.3.4 Correlations between rumen fermentation parameters and bacterial genus composition

The correlation between the composition of ruminal bacteria and rumen fermentation parameters provides valuable insights into their roles in the rumen. The dominant genera that were highly abundant in the supplemented groups showed a significant and positive correlation with nutrient disappearance. Specifically, *Prevotella, Selenomonas*, and *RFN20* were among the genera that were greatly increased with YC and OA supplementation and showed significant medium positive correlations with IVDMD in diet 1. Similarly, a report showed that *Prevotella* was positively correlated with digestibility (66). Another study also reported a positive correlation between *Selenomonas* and DM digestibility (67). In diet 2, *Olsenella, Desulfovibrio*, and *Bifidobacterium* showed a significant positive association with the IVNDFD. The IVCPD showed a significant medium positive correlation with *Succiniclasticum* and *Desulfovibrio* genera. The dominant genera also showed a positive correlation with the rumen fermentation parameters. The *Succiniclasticum, Treponema*, *Desulfovibrio*, and *Sharpea* genera showed a highly significant strong positive correlation with pH. Hence, these genera might be responsible for increased ruminal pH by facilitating fiber digestion. *Succiniclasticum dextrinosolvens* and *Succinimonas amylolytica* are prominent species that play a significant role in starch digestion and are responsible for succinate production. This succinate can then be transformed into propionate through the activity of *Selenomonas ruminantium* (68). *Treponema* enhances cellulose and hemicellulose breakdown (69). NH_3_-N had a significant and strong positive correlation with the *Olsenella, Bulleidia*, and *Oscillospira* genera. However, it showed a significantly strong negative correlation with the *Prevotella, Shuttleworthia*, and *Bifidobacterium* genera. This correlation analysis showed that changes in the composition of the bacterial community may be responsible for the improved disappearance of nutrients and the production of rumen fermentation products including NH_3_-N concentration and volatile fatty acids.

## 5 Conclusion

Taken together, this study showed that the addition of yeast culture, oxalic acid, or both to hemicellulosic diets can increase the disappearance of dry matter, neutral detergent fiber, and crude protein in the rumen. This supplementation also leads to significant improvement in ruminal pH levels, ammonia nitrogen concentration, acetic acid, propionic acid, and TVFA production. However, it caused a reduction in the acetic to propionic acid ratio. Furthermore, it induced modifications in the diversity, richness, and relative abundance of rumen bacteria across various taxonomic levels. The dominant bacterial genera showed a strong positive correlation with nutrient disappearance and rumen fermentation parameters. Therefore, YC and OA have a great potential to buffer and create a conducive rumen environment and improve rumen fermentation efficiency and hemicellulose digestion.

## Data availability statement

The data presented in the study has been deposited in the NCBI Sequence Read Archive (SRA) database (Bio project ID: PRJNA1054784 and PRJNA1054794).

## Ethics statement

The Institutional Animal Care Committee of Jilin Agricultural University (JLAU-ACUC2023-003) approved the comprehensive process used in the management of the experimental animals. The studies were conducted in accordance with the local legislation and institutional requirements. Written informed consent was obtained from the owners for the participation of their animals in this study.

## Author contributions

NA: Data curation, Formal analysis, Methodology, Software, Writing – original draft, Writing – review & editing. LZ: Data curation, Methodology, Writing – original draft. ZW: Data curation, Methodology, Writing – original draft, Software. YX: Data curation, Methodology, Writing – original draft, Software. GY: Data curation, Methodology, Writing – original draft. JD: Data curation, Methodology, Writing – original draft, Software. YZ: Conceptualization, Funding acquisition, Project administration, Resources, Supervision, Validation, Visualization, Writing – review & editing, Data curation. XZ: Conceptualization, Investigation, Resources, Visualization, Writing – review & editing. TW: Conceptualization, Investigation, Resources, Supervision, Validation, Visualization, Writing – review & editing. ZS: Conceptualization, Funding acquisition, Investigation, Project administration, Resources, Supervision, Validation, Writing – review & editing. GQ: Writing – review & editing.

## References

[1] Wang J Zhao G Zhuang Y Chai J Zhang N. Yeast (*Saccharomyces cerevisiae*) culture promotes the performance of fattening sheep by enhancing nutrients digestibility and rumen development. Fermentation. (2022) 8:719. doi: 10.3390/fermentation8120719

[2] Hao X Zhang M Zhang X Mu C Zhang C Zhao J. Effects of feeding corn bran and soybean hulls on nutrient digestibility, rumen microbial protein synthesis, and growth performance of finishing lambs. Animal. (2021) 15:100172. doi: 10.1016/j.animal.2021.100172, PMID: 33589350

[3] Holtzapple M. Hemicelluloses In: Caballero B, editor. Encyclopedia of food science and nutrition. 2nd ed. Texas, USA: Academic Press (2003). 3060–71.

[4] Weimer P. Degradation of cellulose and hemicellulose by ruminal microorganisms. Microorganisms. (2022) 10:2345. doi: 10.3390/microorganisms10122345, PMID: 36557598 PMC9785684

[5] Perdomo MC Marsola RS Favoreto MG Adesogan A Staples CR Santos JEP. Effects of feeding live yeast at 2 dosages on performance and feeding behavior of dairy cows under heat stress. J Dairy Sci. (2020) 103:325–39. doi: 10.3168/jds.2019-17303, PMID: 31677835

[6] Wang X Li F Zhang N Ungerfeld E Guo L Zhang X. Effects of supplementing a yeast culture in a pelleted total mixed ration on fiber degradation, fermentation parameters, and the bacterial community in the rumen of sheep. Anim Feed Sci Technol. (2023) 296:115565. doi: 10.1016/j.anifeedsci.2022.115565

[7] Liu C Ma N Feng Y Zhou M Li H Zhang X. From probiotics to postbiotics: concepts and applications. Anim ResOne Health. (2023) 1:92–114. doi: 10.1002/aro2.7

[8] Majewska MP Miltko R Bełżecki G Kowalik B. Population of protozoa and carbohydrate-digesting enzymes in the rumen of sheep fed a diet supplemented with yeast *Saccharomyces cerevisiae*. Small Rumin Res. (2021) 205:106544. doi: 10.1016/j.smallrumres.2021.106544

[9] Shurson GC. Yeast and yeast derivatives in feed additives and ingredients: sources, characteristics, animal responses, and quantification methods. Anim Feed Sci Technol. (2018) 235:60–76. doi: 10.1016/j.anifeedsci.2017.11.010

[10] Plata F Bárcena-Gama J. Effect of a yeast culture (*Saccharomyces cerevisiae*) on neutral detergent fiber digestion in steers fed oat straw based diets. Anim Feed Sci Technol. (1994) 49:203–10. doi: 10.1016/0377-8401(94)90046-9

[11] Dawson KA Newman KE Boling JA. Effects of microbial supplements containing yeast and lactobacilli on roughage-fed ruminal microbial activities. J Anim Sci. (1990) 68:3392–8. doi: 10.2527/1990.68103392x, PMID: 2123850

[12] Lascano GJ Heinrichs AJ Tricarico JM. Substitution of starch by soluble fiber and *Saccharomyces cerevisiae* dose response on nutrient digestion and blood metabolites for precision-fed dairy heifers. J Dairy Sci. (2012) 95:3298–309. doi: 10.3168/jds.2011-5047, PMID: 22612963

[13] Halfen J Carpinelli N Del Pino FAB Chapman JD Sharman ED Anderson JL. Effects of yeast culture supplementation on lactation performance and rumen fermentation profile and microbial abundance in mid-lactation Holstein dairy cows. J Dairy Sci. (2021) 104:11580–92. doi: 10.3168/jds.2020-19996, PMID: 34454748

[14] Duncan AJ Frutos P Young S. Rates of oxalic acid degradation in the rumen of sheep and goats in response to different levels of oxalic acid administration. Anim Sci. (1997) 65:451–5. doi: 10.1017/S135772980000864X

[15] Strobel BW. Influence of vegetation on low-molecular-weight carboxylic acids in soil solution—A review. Geoderma. (2001) 99:169–98. doi: 10.1016/S0016-7061(00)00102-6

[16] Khampa S Wanapat MJ. Manipulation of rumen fermentation with organic acids supplementation in ruminants raised in the tropics. Pak J Nutr. (2007) 6:20–7. doi: 10.3923/pjn.2007.20.27

[17] Palmieri F Estoppey A House GL Lohberger A Bindschedler S Chain PSG. Oxalic acid, a molecule at the crossroads of bacterial-fungal interactions. Adv Appl Microbiol. (2019) 106:49–77. doi: 10.1016/bs.aambs.2018.10.001 30798804

[18] Benbati M Belenguer A Hervás G Toral P Frutos P Méditerranéens S. Effect of oxalic acid on rumen function and microbiota in sheep fed a low quality diet In: Ben SH, editor. Options Méditerranéennes: Série A. Séminaires Méditerranéens: CIHEAM (2013). 133–8.

[19] Rahman MM Nakagawa T Niimi M Fukuyama K Kawamura O. Effects of feeding oxalate containing grass on intake and the concentrations of some minerals and parathyroid hormone in blood of sheep. Asian Australas J Anim Sci. (2011) 24:940–5. doi: 10.5713/ajas.2011.10445

[20] Chen X. Study on the effective substance of yeast culture regulating rumen function of sheep. [Dissertation]: Jilin Agricultural University, Changchun, China; (2022).

[21] Ruminants NRC. Nutrient requirements of small ruminants: Sheep, goats, cervids, and new world camelids. USA: The National Academies Press, Washington DC (2007).

[22] AOAC. Association of Official Analytical Chemists. Official methods of analysis. Association of Official Agricultural Chemists (USA), Washington, DC (1990). 881–882.

[23] Menke K. Estimation of the energetic feed value obtained from chemical analysis and in vitro gas production using rumen fluid. Anim Res Dev. (1988) 28:7–55.

[24] Huang Z Urriola PE Shurson GC. Use of in vitro dry matter digestibility and gas production to predict apparent total tract digestibility of total dietary fiber for growing pigs. J Anim Sci. (2017) 95:5474–84. doi: 10.2527/jas2017.1964, PMID: 29293750 PMC6292337

[25] DeFeo ME Shampoe KV Carvalho PHV Silva FAS Felix TL. In vitro and in situ techniques yield different estimates of ruminal disappearance of barley. Transl Anim Sci. (2020) 4:141–8. doi: 10.1093/tas/txz170, PMID: 32704974 PMC7200464

[26] Chaney AL Marbach EP. Modified reagents for determination of urea and ammonia. Clin Chem. (1962) 8:130–2. doi: 10.1093/clinchem/8.2.130, PMID: 13878063

[27] Kozich JJ Westcott SL Baxter NT Highlander SK Schloss PD. Development of a dual-index sequencing strategy and curation pipeline for analyzing amplicon sequence data on the MiSeq Illumina sequencing platform. Appl Environ Microbiol. (2013) 79:5112–20. doi: 10.1128/AEM.01043-13, PMID: 23793624 PMC3753973

[28] Nelson A Stewart CJ. Microbiota analysis using sequencing by synthesis: from library preparation to sequencing. Methods Mol Biol. (2020) 2121:165–84. doi: 10.1007/978-1-0716-0338-3_15 32147795

[29] Chao A. Nonparametric estimation of the number of classes in a population. Scand J Stat. (1984) 11:265–70.

[30] Shannon CE. A mathematical theory of communication. Bell Syst Tech J. (1948) 27:379–423. doi: 10.1002/j.1538-7305.1948.tb01338.x, PMID: 38177751

[31] Simpson EH. Measurement of diversity. Nature. (1949) 163:688. doi: 10.1038/163688a0

[32] R Core Team. R: A language and environment for statistical computing. Vienna, Austria: R Foundation for Statistical Computing. (2018).

[33] Habeeb AA. Current view of the significance of yeast for ruminants a review 1-role of yeast and modes of action. Am J Inf Sci Technol. (2017) 1:8–14. doi: 10.11648/j.ajist.20170101.12

[34] Callaway E Martin SA. Effects of a *Saccharomyces cerevisiae* culture on ruminal bacteria that utilize lactate and digest cellulose. J Dairy Sci. (1997) 80:2035–44. doi: 10.3168/jds.S0022-0302(97)76148-4, PMID: 9313145

[35] Dias da Silva A Guedes C Gomes M Loureiro N Brizida E Mena E. Effects of saccharomyces cerevisciae yeast (CNCM I-1077) on ruminal fermentation and fibre degreadation. Proceedings of the International Symposium for Centre for Animal Nutrition; Role of plant cell walls in dairy cow nutrition; (2010).

[36] Zhang J He H Yuan Y Wan K Li L Liu A. Effects of yeast culture supplementation on growth performance, nutrient digestibility, blood metabolites, and immune response in geese. Animals. (2022) 12:1270. doi: 10.3390/ani12101270 35625116 PMC9137895

[37] Rahman MM Rahman MR Niimi M Khadijah WEW Akashi R Abdullah RJ. Effects of different levels of oxalic acid administration on feed intake and nutrient digestibility in goats. Sains Malays. (2017) 46:515–9. doi: 10.17576/jsm-2017-4604-01

[38] Natnael DA Tao W Gui-Xin Q Yu-Guo Z Xue-Feng Z Xue C. Effects of physically effective fiber on rumen and milk parameters in dairy cows: a review. Indian J Anim Res. (2020) 54:1317–23. doi: 10.18805/ijar.B-1104

[39] Abu El-Kassim M Abdou S Hassan E Abdullah MJ. Effect of macroalgae and yeast culture on body performance, blood metabolites, ruminal fermentation and digestibility coefficients of Ossimi lambs. Arch Agri Sci J. (2021) 4:156–67. doi: 10.21608/aasj.2021.77678.1065

[40] Maamouri O Ben SM. Effect of yeast culture feed supply on growth, ruminal pH, and digestibility of fattening calves. Food Sci Nutr. (2021) 9:2762–7. doi: 10.1002/fsn3.2238, PMID: 34026089 PMC8116830

[41] Belenguer A Ben Bati M Hervas G Toral PG Yanez-Ruiz DR Frutos P. Impact of oxalic acid on rumen function and bacterial community in sheep. Animal. (2013) 7:940–7. doi: 10.1017/S1751731112002455, PMID: 23298534

[42] Shen J Zheng W Xu Y Yu Z. The inhibition of high ammonia to in vitro rumen fermentation is pH dependent. Front Vet Sci. (2023) 10:1163021. doi: 10.3389/fvets.2023.1163021 37065225 PMC10097989

[43] Habeeb AA. Importance of yeast in ruminants feeding on production and reproduction. Ecol Evol Biol. (2017) 2:49. doi: 10.11648/j.eeb.20170204.11

[44] Hernández R González SS Pinos-Rodríguez JM Ortega ME Hernández A Bueno G. Effect of a yeast culture on nitrogen balance and digestion in lambs fed early and mature orchard grass. J Appl Anim Res. (2009) 35:53–6. doi: 10.1080/09712119.2009.9706984

[45] Marden J-P Julien C Monteils V Auclair E Moncoulon R Bayourthe C. How does live yeast differ from sodium bicarbonate to stabilize ruminal pH in high-yielding dairy cows? J Dairy Sci. (2008) 91:3528–35. doi: 10.3168/jds.2007-0889, PMID: 18765611

[46] Bryant RW Burns ER Feidler-Cree C Carlton D Flythe MD Martin LJ. Spent craft Brewer’s yeast reduces production of methane and ammonia by bovine rumen microbes. Fanim. (2021) 2:720646. doi: 10.3389/fanim.2021.720646

[47] Nozière P Glasser F Sauvant D. In vivo production and molar percentages of volatile fatty acids in the rumen: a quantitative review by an empirical approach. Animal. (2011) 5:403–14. doi: 10.1017/S1751731110002016 22445407

[48] Liu Y-z Chen X Zhao W Lang M Zhang X-f Wang T. Effects of yeast culture supplementation and the ratio of non-structural carbohydrate to fat on rumen fermentation parameters and bacterial-community composition in sheep. Anim Feed Sci Technol. (2019) 249:62–75. doi: 10.1016/j.anifeedsci.2019.02.003

[49] Amin AB Mao S. Influence of yeast on rumen fermentation, growth performance and quality of products in ruminants: a review. Anim Nutr. (2021) 7:31–41. doi: 10.1016/j.aninu.2020.10.005, PMID: 33997329 PMC8110857

[50] Zhou M Chen Y Guan L. Rumen bacteria In: Puniya A Singh R Kamra D, editors. Rumen microbiology: From evolution to revolution. New Delhi: Springer (2015). 79–95.

[51] Mahayri TM Fliegerová KO Mattiello S Celozzi S Mrázek J Mekadim C. Host species affects bacterial evenness, but not diversity: comparison of fecal bacteria of cows and goats offered the same diet. Animals. (2022) 12:2011. doi: 10.3390/ani12162011, PMID: 36009603 PMC9404439

[52] Zhang Y Zhao J Qin Y Wang Y Yu Z Ning X. Specific alterations of gut microbiota in patients with membranous nephropathy: a systematic review and meta-analysis. Front Physiol. (2022) 13:909491. doi: 10.3389/fphys.2022.909491, PMID: 36388089 PMC9664147

[53] Saleh ASM Zhang Q Chen J Shen Q. Millet grains: nutritional quality, processing, and potential health benefits. Compr Rev Food Sci F. (2013) 12:281–95. doi: 10.1111/1541-4337.12012

[54] Pinnell L Reyes A Wolfe C Weinroth M Metcalf J Delmore R. Bacteroidetes and firmicutes drive differing microbial diversity and community composition between micro-environments in the bovine rumen. Front Vet Sci. (2022) 9:897996. doi: 10.3389/fvets.2022.897996, PMID: 35664853 PMC9161295

[55] Zhang Y Zhang X Li F Li C Li G Zhang D. Characterization of the rumen microbiota and its relationship with residual feed intake in sheep. Animal. (2021) 15:100161. doi: 10.1016/j.animal.2020.100161 33785185

[56] Mizrahi I Wallace RJ Morais S. The rumen microbiome: balancing food security and environmental impacts. Nat Rev Microbiol. (2021) 19:553–66. doi: 10.1038/s41579-021-00543-6, PMID: 33981031

[57] Gharechahi J Vahidi MF Sharifi G Ariaeenejad S Ding XZ Han JL. Lignocellulose degradation by rumen bacterial communities: new insights from metagenome analyses. Environ Res. (2023) 229:115925. doi: 10.1016/j.envres.2023.115925, PMID: 37086884

[58] Palevich N Kelly WJ Ganesh S Rakonjac J Attwood GT. *Butyrivibrio hungatei* MB2003 competes effectively for soluble sugars released by *Butyrivibrio proteoclasticus* B316(T) during growth on Xylan or pectin. Appl Environ Microbiol. (2019) 85:e02056–18. doi: 10.1128/AEM.02056-18 30478228 PMC6344614

[59] Kovár L Savka O Dušková D Marounek M. Fermentation of glucose and xylose in *Prevotella ruminicola* AR29. J Anim Feed Sci. (1999) 8:115–22. doi: 10.22358/jafs/68814/1999

[60] Emerson EL Weimer PJ. Fermentation of model hemicelluloses by Prevotella strains and *Butyrivibrio fibrisolvens* in pure culture and in ruminal enrichment cultures. Appl Microbiol Biotechnol. (2017) 101:4269–78. doi: 10.1007/s00253-017-8150-7, PMID: 28180916

[61] Al-Khaldi SF Durocher LL Martin SA. Deoxyribonuclease activity in *Selenomonas ruminantium*, Streptococcus bovis, and *Bacteroides ovatus*. Curr Microbiol. (2000) 41:182–6. doi: 10.1007/s002840010115, PMID: 10915204

[62] Zhang K Dong X. *Selenomonas bovis* sp. nov., isolated from yak rumen contents. Int J Syst Evol Microbiol. (2009) 59:2080–3. doi: 10.1099/ijs.0.007641-0, PMID: 19605710

[63] Asanuma N Yokoyama S Hino T. Effects of nitrate addition to a diet on fermentation and microbial populations in the rumen of goats, with special reference to S elenomonas ruminantium having the ability to reduce nitrate and nitrite. Anim Sci J. (2015) 86:378–84. doi: 10.1111/asj.12307, PMID: 25439583

[64] Christodoulou C Mavrommatis A Loukovitis D Symeon G Dotas V Kotsampasi B. Effect of Spirulina Dietary Supplementation in Modifying the Rumen Microbiota of Ewes. Animals. (2023) 13:740. doi: 10.3390/ani13040740 36830527 PMC9952741

[65] Hobson PN Mann SO Smith W. Growth factors for *Selenomonas ruminantium*. Nature. (1963) 198:213. doi: 10.1038/198213a0, PMID: 13954887

[66] Li S Zeng H Wang C Han Z. Effect of methionine hydroxy Analog on hu sheep digestibility, rumen fermentation, and rumen microbial community in vitro. Meta. (2023) 13:169. doi: 10.3390/metabo13020169, PMID: 36837788 PMC9968006

[67] Sawanon S Koike S Kobayashi Y. Evidence for the possible involvement of *Selenomonas ruminantium* in rumen fiber digestion. FEMS Microbiol Lett. (2011) 325:170–9. doi: 10.1111/j.1574-6968.2011.02427.x, PMID: 22092507

[68] Kumar S Treloar BP Teh KH McKenzie CM Henderson G Attwood GT. Sharpea and Kandleria are lactic acid producing rumen bacteria that do not change their fermentation products when co-cultured with a methanogen. Anaerobe. (2018) 54:31–8. doi: 10.1016/j.anaerobe.2018.07.008, PMID: 30055268

[69] Kudo H Cheng K-J Costerton J. Interactions between treponema bryantii and cellulolytic bacteria in the in vitro degradation of straw cellulose. Can J Microbiol. (1987) 33:244–8. doi: 10.1139/m87-041, PMID: 3567744

