# Supplementary material for: Effects of yeast culture and oxalic acid supplementation on in vitro nutrient disappearance, rumen fermentation, and bacterial community composition
Source: Front Vet Sci. 2024 Jan 19;10:1330841. doi: 10.3389/fvets.2023.1330841 (PMC10834634; doi:10.3389/fvets.2023.1330841)
Supplement: Supplementary file 1 [file Data_Sheet_1.docx]

Supplementary Material

**Yeast culture and oxalic acid supplementation improve sheep rumen fermentation efficiency and bacterial community composition*:* in vitro**

**Natnael D. Aschalew^1, 2^, Longyu Zhang^1^，Ziyuan Wang^1^, Yuanhong Xia^1^，Guopei Yin^1^, Jianan Dong^1^, Yuguo Zhen^1, 4^, Xuefeng Zhang^1, 4^, Tao Wang**^1^**^, 4^, Zhe Sun^1, 3, 4*^, Guixin Qin^1, 4*^**

* Corresponding authors:

Email addresses: [sunzhe198615@163.com](mailto:sunzhe198615@163.com) (Z. Sun)，[qgx@jlau.edu.cn](mailto:qgx@jlau.edu.cn) (G.-x. Qin).

Present address: At Jilin Agricultural University, Changchun, 130118, P.R. China.

Supplementary Figures and Tables


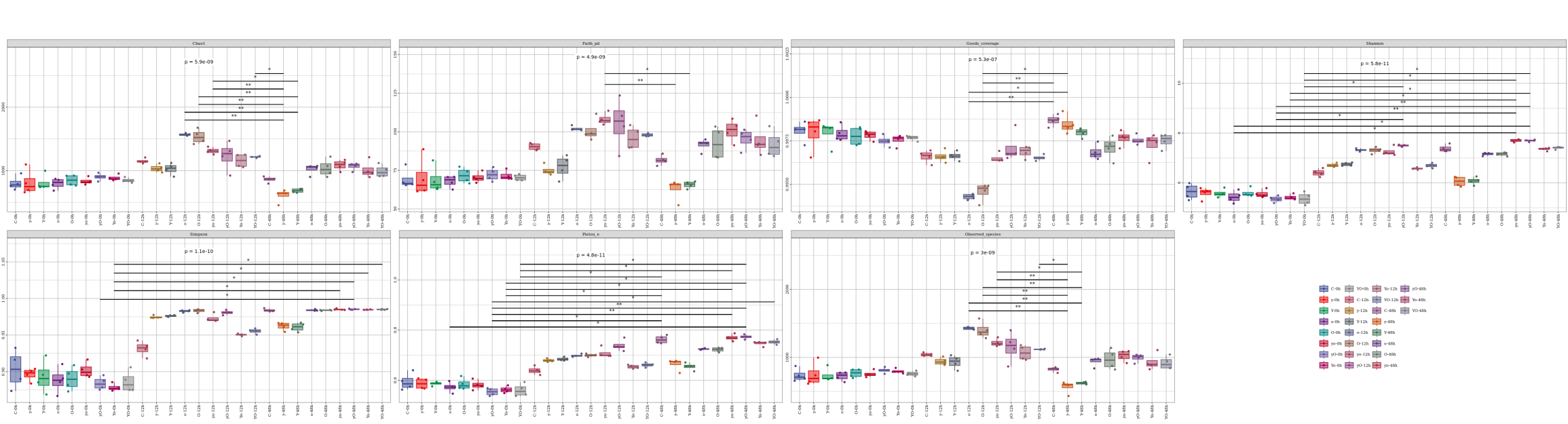


**Supplementary Figure 1**. The alpha diversity of the dominant bacteria species in diet 1 at different fermentation times.

Where: C = Diet 1 (10.3% hemicellulose); y = diet 1 with low yeast culture; Y =Diet 1 with high yeast culture; o = diet 1 with low oxalic acid; O = diet 1 with high oxalic acid; yo = diet 1 with low yeast culture and low oxalic acid; yO = diet 1 with low yeast culture and high oxalic acid; Yo = diet 1 with high yeast culture and low oxalic acid; YO = diet 1 with high yeast culture and high oxalic acid. The abscissa is the name of each experimental treatment and the ordinate is the relative abundance of each phylum at a specific fermentation time. Each panel corresponds to an alpha diversity index, which is identified in a gray area at the top. In each panel, the abscissa is the grouping label, and the ordinate is the value of the corresponding alpha diversity index. In the box plot, the symbols have the following meanings: the upper and lower end lines of the box, the upper and lower quartile ranges (IQR); median, median; upper and lower edges, maximum and minimum values (extrema within the IQR range of 1.5 times); A dot on the outside of the upper and lower edges, indicating an outlier. The numbers under the Diversity Index label are the *p*-values of the Kruskal-Wallis test. When two groups are compared, the explicit marker of Dunn's test is drawn by default.


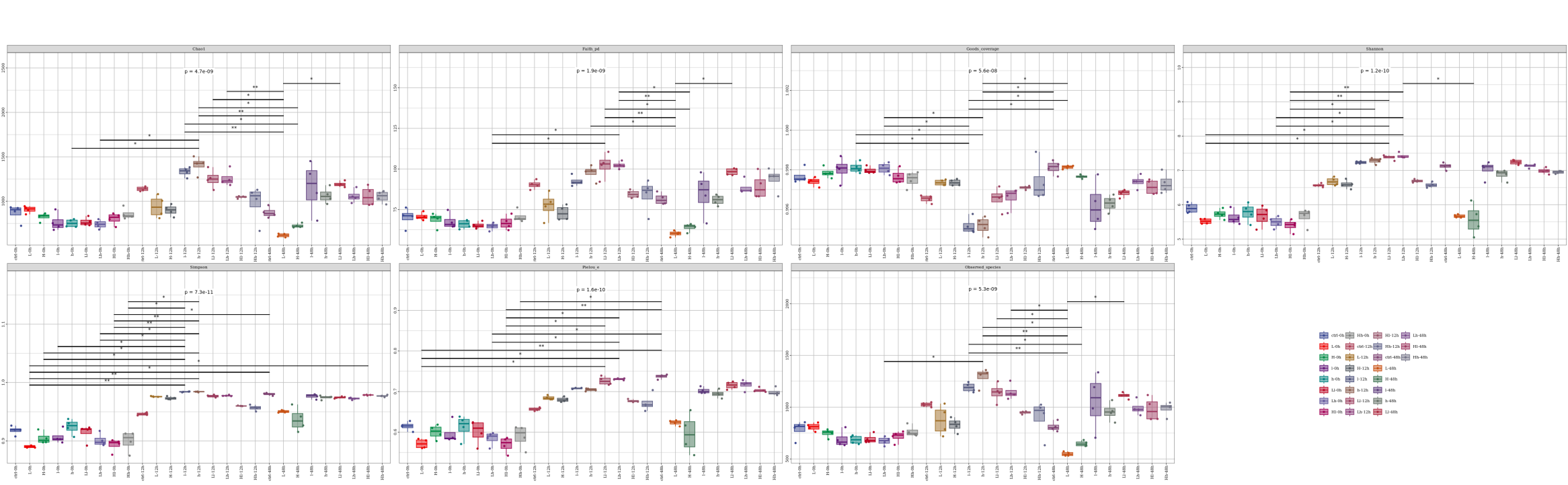


**Supplementary Figure 2**. The alpha diversity of the dominant bacteria species in diet 2 at different fermentation times.

Where: ctrl = Diet 2 (17% hemicellulose); L = diet 2 with low yeast culture; H = diet 2 with high yeast culture; l = diet 2 with low oxalic acid; h = diet 2 with high oxalic acid; Ll = diet 2 with low yeast culture and low oxalic acid; Lh = diet 2 with low yeast culture and high oxalic acid; Hl = diet 2 with high yeast culture and low oxalic acid; Hh = diet 2 with high yeast culture and high oxalic acid. The abscissa is the name of each experimental treatment and the ordinate is the relative abundance of each phylum at a specific fermentation time. Each panel corresponds to an alpha diversity index, which is identified in a gray area at the top. In each panel, the abscissa is the grouping label, and the ordinate is the value of the corresponding alpha diversity index. In the box plot, the symbols have the following meanings: the upper and lower end lines of the box, the upper and lower quartile ranges (IQR); median, median; upper and lower edges, maximum and minimum values (extrema within the IQR range of 1.5 times); A dot on the outside of the upper and lower edges, indicating an outlier. The numbers under the Diversity Index label are the *p*-values of the Kruskal-Wallis test. When two groups are compared, the explicit marker of Dunn's test is drawn by default.

**Supplementary Table 1**. Effects of supplementation of YC and OA in diet 1 on ruminal bacteria genera composition (%).

| Genus | Time | Diet 1 | Yeast culture (YC) | | Oxalic acid (OA) | | Interactions | | | | *SEM* | *P* values | | |
| --- | --- | --- | --- | --- | --- | --- | --- | --- | --- | --- | --- | --- | --- | --- |
|  |  | C | y | Y | o | O | yo | yO | Yo | YO |  | YC | OA | YC × OA |
| *Prevotella* | 0 h | 49.12 | 52.77 | 51.66 | 52.79 | 54.50 | 47.85 | 52.33 | 57.84 | 53.71 | 0.88 | 0.265 | 0.523 | 0.298 |
|  | 12 h | 57.99^e^ | 27.53^bc^ | 27.98^c^ | 48.03^d^ | 46.75^d^ | 21.40^a^ | 21.83^ab^ | 46.99^d^ | 43.69^d^ | 2.17 | <0.001 | 0.386 | <0.001 |
|  | 48 h | 3.43^a^ | 16.52^c^ | 16.92^c^ | 24.89^d^ | 26.45^d^ | 5.27^ab^ | 5.37^ab^ | 8.61^b^ | 9.99^b^ | 1.40 | <0.001 | 0.159 | <0.001 |
| *Butyrivibrio* | 0 h | 10.66^b^ | 8.64^ab^ | 9.30^ab^ | 7.17^ab^ | 6.92^a^ | 9.05^ab^ | 9.14^ab^ | 6.67^a^ | 8.97^ab^ | 0.31 | 0.495 | 0.021 | 0.035 |
|  | 12 h | 2.40^a^ | 4.65^d^ | 4.64^d^ | 2.68^ab^ | 2.41^a^ | 3.38^bc^ | 3.92^cd^ | 2.79^ab^ | 2.99^ab^ | 0.15 | <0.001 | <0.001 | <0.001 |
|  | 48 h | 9.70^c^ | 3.57^ab^ | 3.48^ab^ | 2.48^a^ | 2.58^a^ | 3.10^a^ | 3.54^ab^ | 5.15^b^ | 5.06^b^ | 0.37 | <0.001 | <0.001 | <0.001 |
| *Selenomonas* | 0 h | 0.01^ab^ | 0.02^ab^ | 0.02^ab^ | 0.03^b^ | 0.02^ab^ | 0.01^ab^ | 0.01^ab^ | 0.00^a^ | 0.01^ab^ | 0.002 | 0.067 | 0.212 | 0.016 |
|  | 12 h | 0.00^a^ | 0.21^b^ | 0.15^ab^ | 0.48^c^ | 0.48^c^ | 0.10^ab^ | 0.16^ab^ | 0.08^ab^ | 0.06^ab^ | 0.03 | <0.001 | 0.002 | <0.001 |
|  | 48 h | 1.32^a^ | 24.55^c^ | 23.53^c^ | 4.18^ab^ | 4.29^ab^ | 6.98^ab^ | 7.94^b^ | 5.96^ab^ | 5.42^ab^ | 1.41 | <0.001 | <0.001 | <0.001 |
| *Succiniclasticum* | 0 h | 0.45^ab^ | 0.39^ab^ | 0.58^b^ | 0.23^ab^ | 0.30^ab^ | 0.38^ab^ | 0.29^ab^ | 0.14^ab^ | 0.12^a^ | 0.03 | 0.488 | 0.001 | 0.064 |
|  | 12 h | 0.47^ab^ | 0.15^a^ | 0.04^a^ | 6.26^e^ | 7.92^e^ | 4.20^d^ | 3.52^cd^ | 0.94^ab^ | 2.16^bc^ | 0.47 | <0.001 | <0.001 | <0.001 |
|  | 48 h | 3.01^a^ | 2.79^a^ | 2.65^a^ | 9.20^c^ | 8.52^c^ | 6.41^b^ | 5.81^b^ | 9.98^c^ | 8.81^c^ | 0.48 | <0.001 | <0.001 | <0.001 |
| *Ruminococcaceae_Ruminococcus* | 0 h | 4.78^a^ | 4.50^a^ | 4.86^a^ | 1.17^b^ | 8.75^ab^ | 7.64^ab^ | 6.98^ab^ | 8.95^ab^ | 5.25^a^ | 0.53 | 0.086 | <0.001 | 0.412 |
|  | 12 h | 1.12^e^ | 0.51^bc^ | 0.76^cd^ | 0.19^a^ | 0.17^a^ | 0.35^ab^ | 0.43^ab^ | 0.94^de^ | 0.72^cd^ | 0.06 | <0.001 | <0.001 | <0.001 |
|  | 48 h | 0.65^c^ | 0.35^ab^ | 0.33^ab^ | 0.30^a^ | 0.29^a^ | 0.38^ab^ | 0.37^ab^ | 0.50^bc^ | 0.45^ab^ | 0.02 | 0.117 | 0.073 | <0.001 |
| *Shuttleworthia* | 0 h | 0.11^ab^ | 0.11^ab^ | 0.20^b^ | 0.21^b^ | 0.21^b^ | 0.10^ab^ | 0.05^a^ | 0.11^ab^ | 0.04^a^ | 0.01 | 0.004 | 0.212 | 0.006 |
|  | 12 h | 0.01^a^ | 1.41^b^ | 2.20^c^ | 3.11^d^ | 4.00^e^ | 0.30^a^ | 0.40^a^ | 0.02^a^ | 0.02^a^ | 0.24 | <0.001 | 0.004 | <0.001 |
|  | 48 h | 0.21^a^ | 0.75^a^ | 0.79^a^ | 2.91^b^ | 2.81^b^ | 0.32^a^ | 0.55^a^ | 0.42^a^ | 0.37^a^ | 0.18 | <0.001 | <0.001 | <0.001 |
| *Olsenella* | 0 h | 0.95 | 1.02 | 1.00 | 1.09 | 1.20 | 1.00 | 1.07 | 1.10 | 0.79 | 0.04 | 0.523 | 0.747 | 0.312 |
|  | 12 h | 0.25^a^ | 1.25^bc^ | 1.19^b^ | 0.40^a^ | 0.37^a^ | 1.83^d^ | 1.67^cd^ | 1.02^b^ | 0.99^b^ | 0.09 | <0.001 | 0.063 | 0.005 |
|  | 48 h | 0.34^a^ | 1.28^b^ | 1.37^b^ | 0.29^a^ | 0.29^a^ | 2.34^c^ | 2.49^c^ | 1.20^b^ | 1.34^b^ | 0.13 | <0.001 | <0.001 | <0.001 |

**Supplementary Table 1.** (*continued*)

| Genus | Time | Diet 1 | Yeast culture (YC) | | Oxalic acid (OA) | | Interactions | | | | *SEM* | *P* values | | |
| --- | --- | --- | --- | --- | --- | --- | --- | --- | --- | --- | --- | --- | --- | --- |
|  |  | C | y | Y | o | O | yo | yO | Yo | YO |  | YC | OA | YC × OA |
| *Bulleidia* | 0 h | 0.49^b^ | 0.42^ab^ | 0.47^b^ | 0.35^ab^ | 0.38^ab^ | 0.39^ab^ | 0.28^a^ | 0.26^a^ | 0.29^a^ | 0.02 | 0.128 | <0.001 | 0.12 |
|  | 12 h | 0.30^a^ | 1.23^c^ | 1.24^c^ | 0.21^a^ | 0.22^a^ | 0.75^b^ | 0.97^bc^ | 1.02^bc^ | 0.98^bc^ | 0.07 | <0.001 | 0.003 | 0.250 |
|  | 48 h | 1.96^d^ | 1.87^cd^ | 1.88^cd^ | 0.70^a^ | 0.75^a^ | 1.22^ab^ | 1.32^abc^ | 1.86^bcd^ | 1.85^bcd^ | 0.09 | <0.001 | <0.001 | <0.001 |
| *Treponema* | 0 h | 0.95^b^ | 0.80^ab^ | 0.67^ab^ | 0.44^a^ | 0.55^ab^ | 0.70^ab^ | 0.53^a^b | 0.46^a^ | 0.51^a^ | 0.04 | 0.183 | <0.001 | 0.192 |
|  | 12 h | 1.33^de^ | 0.01^a^ | 0.04^ab^ | 0.54^bc^ | 0.54^abc^ | 0.98^cd^ | 1.34^de^ | 1.50^de^ | 1.68^e^ | 0.15 | 0.005 | <0.001 | <0.001 |
|  | 48 h | 0.47^b^ | 0.03^a^ | 0.04^a^ | 0.70^bc^ | 0.58^b^ | 1.11^d^ | 1.03^cd^ | 0.38^ab^ | 0.65^b^ | 0.06 | <0.001 | <0.001 | <0.001 |
| *Succinivibrio* | 0 h | 0.02 | 0.03 | 0.01 | 0.02 | 0.02 | 0.02 | 0.01 | 0.01 | 0.02 | 0.002 | 0.625 | 0.297 | 0.323 |
|  | 12 h | 0.74^b^ | 0.03^a^ | 0.03^a^ | 0.63^b^ | 0.50^b^ | 0.01^a^ | 0.02^a^ | 0.02^a^ | 0.02^a^ | 0.05 | <0.001 | 0.315 | 0.489 |
|  | 48 h | 0.07^c^ | 0.05^a^ | 0.05^a^ | 3.85^b^ | 4.06^b^ | 0.03^a^ | 0.03^a^ | 0.23^a^ | 0.17^a^ | 0.44 | <0.001 | 0.001 | <0.001 |
| *RFN20* | 0 h | 0.44 | 0.42 | 0.41 | 0.49 | 0.60 | 0.48 | 0.37 | 0.44 | 0.36 | 0.02 | 0.080 | 0.659 | 0.301 |
|  | 12 h | 0.24^a^ | 0.83^e^ | 1.18^f^ | 0.65^cde^ | 0.56^bcde^ | 0.51^abcd^ | 0.73^de^ | 0.38^abc^ | 0.32^ab^ | 0.05 | <0.001 | <0.001 | <0.001 |
|  | 48 h | 0.73^a^ | 1.55^b^ | 1.40^b^ | 1.36^b^ | 1.52b | 0.67^a^ | 0.72^a^ | 0.56^a^ | 0.89^a^ | 0.07 | <0.001 | <0.001 | <0.001 |
| *Desulfovibrio* | 0 h | 0.03^a^ | 0.03^a^ | 0.04^ab^ | 0.06^ab^ | 0.09^b^ | 0.02^a^ | 0.02^a^ | 0.02^a^ | 0.03^a^ | 0.01 | 0.002 | 0.367 | 0.034 |
|  | 12 h | 0.11^a^ | 0.03^a^ | 0.04^a^ | 1.00^b^ | 1.16^b^ | 0.03^a^ | 0.08^a^ | 0.02^a^ | 0.01^a^ | 0.08 | <0.001 | <0.001 | <0.001 |
|  | 48 h | 0.96^a^ | 1.21^a^ | 1.00^a^ | 3.71^b^ | 3.04^ab^ | 2.04^ab^ | 1.94^ab^ | 3.70^b^ | 4.05^b^ | 0.24 | 0.012 | <0.001 | 0.094 |
| *Sharpea* | 0 h | 0.42 | 0.50 | 0.66 | 0.42 | 0.83 | 0.37 | 0.16 | 0.24 | 0.11 | 0.06 | 0.183 | 0.330 | 0.059 |
|  | 12 h | 0.07^a^ | 0.32^a^ | 0.35^a^ | 0.30^a^ | 0.27^a^ | 2.82^b^ | 3.15^b^ | 0.32^a^ | 0.43^a^ | 0.20 | <0.001 | <0.001 | <0.001 |
|  | 48 h | 0.13^a^ | 0.09^a^ | 0.10^a^ | 0.58^a^ | 0.65^a^ | 3.10^b^ | 2.41^b^ | 0.46^a^ | 0.40^a^ | 0.19 | <0.001 | <0.001 | <0.001 |
| *Bifidobacterium* | 0 h | 0.22 | 0.20 | 0.25 | 0.34 | 0.27 | 0.26 | 0.22 | 0.26 | 0.22 | 0.01 | 0.201 | 0.054 | 0.593 |
|  | 12 h | 0.03^a^ | 0.73^c^ | 0.79^c^ | 0.97^c^ | 0.92^c^ | 0.36^b^ | 0.35^b^ | 0.14^ab^ | 0.14^ab^ | 0.06 | <0.001 | 0.571 | <0.001 |
|  | 48 h | 0.40^a^ | 1.07^b^ | 1.30^b^ | 1.31^b^ | 1.23^b^ | 0.91^b^ | 0.96^b^ | 1.01^b^ | 1.08^b^ | 0.06 | 0.165 | 0.119 | <0.001 |

^a, b, c, d, e^ Different superscripts in the same raw imply their mean values are significantly different (P ≤ 0.05). Where: C = Diet 1 (10.3% hemicellulose); y = diet 1 with low yeast culture; Y = Diet 1 with high yeast culture; o = diet 1 with low oxalic acid; O = diet 1 with high oxalic acid; yo = diet 1 with low yeast culture and low oxalic acid; yO = diet 1 with low yeast culture and high oxalic acid; Yo = diet 1 with high yeast culture and low oxalic acid; YO = diet 1 with high yeast culture and high oxalic acid.

**Supplementary Table 2**. Effects of supplementation of YC and OA in diet2 on dominant bacteria genera composition (%).

| Genus | Time | Diet 1 | Yeast culture (YC) | | Oxalic acid (OA) | | Interactions | | | | *SEM* | *P* values | | |
| --- | --- | --- | --- | --- | --- | --- | --- | --- | --- | --- | --- | --- | --- | --- |
|  |  | ctrl | L | H | l | h | Ll | Lh | Hl | Hh |  | YC | OA | YC × OA |
| *Prevotella* | 0 h | 49.77^ab^ | 54.89^b^ | 54.37^b^ | 50.53^ab^ | 43.24^a^ | 46.56^ab^ | 53.10^b^ | 55.23^b^ | 55.47^b^ | 0.88 | <0.001 | 0.239 | 0.01 |
|  | 12 h | 53.47^e^ | 29.00^b^ | 26.31^b^ | 47.91^d^ | 48.13^d^ | 20.57^a^ | 21.47^a^ | 42.10^c^ | 41.65^c^ | 2.01 | <0.001 | 0.542 | <0.001 |
|  | 48 h | 4.84^a^ | 14.70^bc^ | 20.84^d^ | 32.20^e^ | 32.12^e^ | 10.43^ab^ | 7.30^a^ | 16.73^cd^ | 15.55^bcd^ | 1.60 | <0.001 | <0.001 | <0.001 |
| *Selenomonas* | 0 h | 0.00^a^ | 0.02^ab^ | 0.01^ab^ | 0.02^b^ | 0.022^b^ | 0.02^b^ | 0.02^b^ | 0.02^b^ | 0.02^b^ | 0.00 | 1.99 | <0.001 | 2.228 |
|  | 12 h | 0.02^a^ | 0.35^bcd^ | 0.37^cd^ | 0.73^e^ | 0.56^de^ | 0.16^abc^ | 0.09^a^ | 0.13^ab^ | 0.07^a^ | 0.04 | <0.001 | 0.021 | <0.001 |
|  | 48 h | 1.58^a^ | 35.52^cd^ | 42.18^d^ | 15.37^ab^ | 16.17^b^ | 24.75^bc^ | 24.70^bc^ | 17.91^b^ | 18.03^b^ | 2.08 | <0.001 | 0.009 | <0.001 |
| *Butyrivibrio* | 0 h | 9.54^ab^ | 8.11^a^ | 8.26^a^ | 10.39^abc^ | 13.68^d^ | 12.85^cd^ | 12.22^bcd^ | 10.30^abc^ | 8.59^a^ | 0.38 | <0.001 | <0.001 | 0.002 |
|  | 12 h | 3.61^abc^ | 3.93^bc^ | 4.40^c^ | 3.20^ab^ | 3.00^a^ | 3.78^abc^ | 3.62^abc^ | 3.23^ab^ | 3.63^abc^ | 0.09 | 0.004 | <0.001 | 0.106 |
|  | 48 h | 12.17^c^ | 3.61^b^ | 2.04^a^ | 1.35^a^ | 1.52^a^ | 0.85^a^ | 1.18^a^ | 1.20^a^ | 1.24^a^ | 0.58 | <0.001 | <0.001 | <0.001 |
| *Succiniclasticum* | 0 h | 0.14 | 0.23 | 0.14 | 0.11 | 0.13 | 0.18 | 0.23 | 0.07 | 0.22 | 0.02 | 0.099 | 0.203 | 0.717 |
|  | 12 h | 0.94^ab^ | 0.07^a^ | 0.07^a^ | 6.10^c^ | 5.85^c^ | 2.50^b^ | 1.92^ab^ | 1.17^ab^ | 1.47^ab^ | 0.38 | <0.001 | <0.001 | <0.001 |
|  | 48 h | 1.57^a^ | 1.42^a^ | 1.25^a^ | 5.48^b^ | 5.66^b^ | 5.31^b^ | 7.07^b^ | 13.09^c^ | 13.04^c^ | 0.74 | <0.001 | <0.001 | <0.001 |
| *Ruminococcaceae_Ruminococcus* | 0 h | 6.46 | 5.45 | 7.24 | 6.07 | 5.77 | 5.31 | 3.01 | 2.72 | 5.44 | 0.46 | 0.396 | 0.239 | 0.332 |
|  | 12 h | 1.23^c^ | 0.86^b^ | 0.80^b^ | 0.26^a^ | 0.26^a^ | 0.51^a^ | 0.46^a^ | 0.82^b^ | 0.88^b^ | 0.05 | <0.001 | <0.001 | <0.001 |
|  | 48 h | 0.55^d^ | 0.26^abc^ | 0.27^bc^ | 0.22^ab^ | 0.22^ab^ | 0.13^a^ | 0.21^ab^ | 0.36^c^ | 0.33^bc^ | 0.02 | <0.001 | <0.001 | <0.001 |
| *Shuttleworthia* | 0 h | 0.14 | 0.10 | 0.13 | 0.15 | 0.20 | 0.25 | 0.10 | 0.04 | 0.18 | 0.02 | 0.657 | 0.785 | 0.203 |
|  | 12 h | 0.00^a^ | 2.45^b^ | 1.82^b^ | 4.99^d^ | 4.06^c^ | 0.49^a^ | 0.64^a^ | 0.02^a^ | 0.02^a^ | 0.30 | <0.001 | 0.022 | <0.001 |
|  | 48 h | 0.29^a^ | 0.29^a^ | 1.41^ab^ | 6.87^d^ | 5.94^cd^ | 3.76^bc^ | 2.03^ab^ | 1.33^ab^ | 0.38^a^ | 0.41 | <0.001 | <0.001 | <0.001 |
|  | 12 h | 0.53^ab^ | 1.54^de^ | 1.86^e^ | 0.32^a^ | 0.28^a^ | 1.10^cd^ | 0.98^a^ | 1.11^cd^ | 1.28^cd^ | 0.09 | <0.001 | <0.001 | 0.114 |
|  | 48 h | 3.95^d^ | 1.55^c^ | 0.98^bc^ | 0.13^a^ | 0.10^a^ | 0.23^ab^ | 0.30^ab^ | 0.29^ab^ | 0.23^ab^ | 0.21 | <0.001 | <0.001 | <0.001 |
| *Olsenella* | 0 h | 1.56^b^ | 0.90^a^ | 1.21^ab^ | 1.12^ab^ | 1.15^ab^ | 1.03^ab^ | 0.73^a^ | 0.87^a^ | 1.21^ab^ | 0.06 | 0.005 | 0.107 | 0.089 |
|  | 12 h | 0.32^a^ | 1.38^bc^ | 1.43^c^ | 0.20^a^ | 0.28^a^ | 1.83^d^ | 1.60^cd^ | 1.02^b^ | 1.03^b^ | 0.10 | <0.001 | 0.549 | <0.001 |
|  | 48 h | 0.57^ab^ | 1.41^c^ | 0.73^bc^ | 0.01^a^ | 0.01^a^ | 0.20^ab^ | 0.23^ab^ | 0.19^ab^ | 0.17^ab^ | 0.08 | 0.008 | <0.001 | 0.115 |

**Supplementary Table 2.** (continued)

| Genus | Time | Diet 2 | Yeast culture (YC) | | Oxalic acid (OA) | | Interactions | | | | *SEM* | *P* values | | |
| --- | --- | --- | --- | --- | --- | --- | --- | --- | --- | --- | --- | --- | --- | --- |
|  |  | ctrl | L | H | l | h | Ll | Lh | Hl | Hh |  | YC | OA | YC × OA |
| *Bulleidia* | 0 h | 0.42^abc^ | 0.24^a^ | 0.34^ab^ | 0.41^abc^ | 0.60^c^ | 0.50^bc^ | 0.35^abc^ | 0.28^ab^ | 0.33^ab^ | 0.02 | 0.003 | 0.105 | 0.011 |
|  | 12 h | 0.53^ab^ | 1.54^de^ | 1.86^e^ | 0.32^a^ | 0.28^a^ | 1.10^cd^ | 0.98^a^ | 1.11^cd^ | 1.28^cd^ | 0.09 | <0.001 | <0.001 | 0.114 |
|  | 48 h | 3.95^d^ | 1.55^c^ | 0.98^bc^ | 0.13^a^ | 0.10^a^ | 0.23^ab^ | 0.30^ab^ | 0.29^ab^ | 0.23^ab^ | 0.21 | <0.001 | <0.001 | <0.001 |
| *Treponema* | 0 h | 0.45^ab^ | 0.56^b^ | 0.47^ab^ | 0.48^ab^ | 0.44^ab^ | 0.40^ab^ | 0.24^a^ | 0.38^ab^ | 0.41^ab^ | 0.02 | 0.486 | 0.035 | 0.87 |
|  | 12 h | 1.37^de^ | 0.04^a^ | 0.02^a^ | 0.68^bc^ | 0.51^ab^ | 1.17^cd^ | 1.73^def^ | 2.31^f^ | 1.80^ef^ | 0.13 | <0.001 | <0.001 | <0.001 |
|  | 48 h | 0.74^b^ | 0.02^a^ | 0.03^a^ | 0.94^b^ | 0.89^b^ | 2.84^c^ | 2.34^c^ | 1.12^b^ | 0.95^b^ | 0.15 | <0.001 | <0.001 | <0.001 |
| *Succinivibrio* | 0 h | 0.01 | 0.02 | 0.02 | 0.02 | 0.01 | 0.01 | 0.02 | 0.02 | 0.01 | 0.00 | 0.876 | 0.359 | 0.191 |
|  | 12 h | 0.28^b^ | 0.05^a^ | 0.04^b^ | 0.68^c^ | 0.56^c^ | 0.01^a^ | 0.01^a^ | 0.01^a^ | 0.02^a^ | 0.04 | <0.001 | 0.002 | <0.001 |
|  | 48 h | 7.74^b^ | 0.05^a^ | 0.04^a^ | 5.25^b^ | 7.84^b^ | 0.02^a^ | 0.02^a^ | 0.11^a^ | 0.07^a^ | 0.60 | <0.001 | 0.248 | 0.215 |
| *RFN20* | 0 h | 0.27 | 0.31 | 0.32 | 0.29 | 0.24 | 0.31 | 0.07 | 0.14 | 0.22 | 0.02 | 0.654 | 0.054 | 0.086 |
|  | 12 h | 0.20^a^ | 1.68^e^ | 1.34^e^ | 0.77^cd^ | 0.73^bcd^ | 0.73^bcd^ | 0.80^d^ | 0.41^abc^ | 0.38^ab^ | 0.08 | <0.001 | <0.001 | <0.001 |
|  | 48 h | 0.63^a^ | 0.98^ab^ | 0.97^ab^ | 1.08^b^ | 1.23^b^ | 1.18^b^ | 0.65^a^ | 1.02^ab^ | 1.03^ab^ | 0.04 | 0.604 | 0.009 | <0.001 |
| *Desulfovibrio* | 0 h | 0.05 | 0.02 | 0.04 | 0.07 | 0.06 | 0.07 | 0.04 | 0.01 | 0.05 | 0.01 | 0.334 | 0.550 | 0.269 |
|  | 12 h | 0.15^a^ | 0.04^a^ | 0.06^a^ | 1.21^b^ | 1.42^b^ | 0.05^a^ | 0.06^a^ | 0.02^a^ | 0.02^a^ | 0.09 | <0.001 | <0.001 | <0.001 |
|  | 48 h | 0.48^abc^ | 0.10^a^ | 0.83^abc^ | 0.26^ab^ | 0.09^a^ | 0.90^bcd^ | 1.12^cd^ | 1.49^d^ | 1.42^d^ | 0.10 | <0.001 | 0.006 | 0.002 |
| *Sharpea* | 0 h | 0.39 | 0.30 | 0.38 | 0.36 | 0.28 | 0.56 | 0.15 | 0.05 | 0.60 | 0.06 | 0.998 | 0.973 | 0.126 |
|  | 12 h | 0.06^a^ | 0.38^a^ | 0.23^a^ | 0.38^a^ | 0.33^a^ | 2.14^b^ | 2.34^b^ | 0.32^a^ | 0.31^a^ | 0.14 | <0.001 | <0.001 | <0.001 |
|  | 48 h | 0.30^ab^ | 0.07^a^ | 0.04^a^ | 0.10^ab^ | 0.07^a^ | 0.11^ab^ | 0.32^b^ | 0.05^a^ | 0.05^a^ | 0.02 | 0.018 | 0.334 | 0.005 |
| *Bifidobacterium* | 0 h | 0.34^ab^ | 0.20^a^ | 0.30^ab^ | 0.35^ab^ | 0.43^b^ | 0.38^ab^ | 0.32^ab^ | 0.21^a^ | 0.29^ab^ | 0.02 | 0.014 | 0.148 | 0.034 |
|  | 12 h | 0.03^a^ | 1.15^cd^ | 1.45^d^ | 0.97^c^ | 1.01^c^ | 0.51^b^ | 0.42^b^ | 0.19^ab^ | 0.22^ab^ | 0.08 | 0.528 | <0.001 | <0.001 |
|  | 48 h | 0.94^bc^ | 1.64^c^ | 0.85^ab^ | 0.15^a^ | 0.16^ab^ | 0.14^a^ | 0.20^ab^ | 0.16^ab^ | 0.14^a^ | 0.10 | 0.094 | <0.001 | 0.102 |

^a, b, c, d, e^ Different superscripts in the same raw imply their mean values are significantly different (*P* ≤ 0.05). Where: ctrl = diet 2 (17% hemicellulose); L = diet 2 with low yeast culture; H = diet 2 with high yeast culture; l = diet 2 with low oxalic acid; h = diet 2 with high oxalic acid; Ll = diet 2 with low yeast culture and low oxalic acid; Lh = diet 2 with low yeast culture and high oxalic acid; Hl = diet 2 with high yeast culture and low oxalic acid; Hh = diet 2 with high yeast culture and high oxalic acid.
